# Hand hygiene intervention to optimize helminth infection control: Design and baseline results of *Mikono Safi*–An ongoing school-based cluster-randomised controlled trial in NW Tanzania

**DOI:** 10.1371/journal.pone.0242240

**Published:** 2020-12-09

**Authors:** Kenneth Makata, Safari Kinung’hi, Christian Hansen, Philip Ayieko, Simon Sichalwe, Onike Mcharo, Jeroen Ensink, Robert Dreibelbis, Sarah Rockowitz, Elialilia Okello, Heiner Grosskurth, Saidi Kapiga

**Affiliations:** 1 Mwanza Intervention Trials Unit, Mwanza, Tanzania; 2 National Institute for Medical Research, Mwanza, Tanzania; 3 MRC Tropical Epidemiology Group, London School of Hygiene and Tropical Medicine, London, United Kingdom; 4 Department of Disease Control, London School of Hygiene and Tropical Medicine, London, United Kingdom; 5 Johns Hopkins Bloomberg School of Public Health, Baltimore, Maryland, United States of America; PLOS ONE, UNITED KINGDOM

## Abstract

**Introduction:**

Soil transmitted helminths (STH) can affect over 50% of children in some parts of Tanzania. Control measures involve annual deworming campaigns in schools, but re-infection is rapid. This paper presents the design and baseline survey results of an ongoing school-based cluster-randomised controlled trial in Kagera region, NW Tanzania. The trial aims to determine whether the effect of routine deworming on the prevalence of *Ascaris lumbricoides* and *Trichuris trichiura* infections among school aged children can be sustained when combined with a behaviour change intervention promoting handwashing with water and soap.

**Methods:**

As part of the trial, a total of 16 schools were randomised to receive the intervention (N = 8) or as controls (N = 8). Randomisation was stratified per district and restricted to ensure pre-trial STH prevalence was balanced between study arms. The combination intervention to be tested comprises class-room based teacher-led health education, improvement of handwash stations, coloured nudges to facilitate handwashing and parental engagement sessions. The impact evaluation involves two cross-sectional surveys conducted at baseline and endline. The objectives of the baseline survey were: (i) to confirm whether the deworming campaign was successful, and identify and treat students still infected about 2 weeks after deworming, (ii) to document any baseline differences in STH prevalence between trial arms, and (iii) to assess handwashing behaviours, and access to water and sanitation at school and home. We randomly sampled 35 students per class in Grades 1–6 (an average of 200 children per school), stratified to ensure equal representation between genders. Assenting students were interviewed using a structured questionnaire and asked to provide a stool specimen.

**Results:**

Results of the baseline survey conducted about 2 weeks after deworming shows balanced demographic and STH prevalence data across trial arms. We observed a low prevalence of ascariasis (< 5%) as expected; however, the prevalence of trichuriasis was still about 35% in both arms.

**Conclusion:**

The randomisation procedure was successful in achieving a balanced distribution of demographic characteristics and helminth infections between trial arms. The intervention is being rolled out. The current deworming treatment regimen may need to be revised with regards to the treatment of trichuriasis.

## Introduction

Soil transmitted helminth (STH) infections frequently affect children in low- and -middle income countries. A low worm load rarely causes health problems, but heavier infections are frequent and are associated with malabsorption of nutrients, anaemia, gastro-intestinal symptoms and general malaise [1]; and may lead to impaired physical development and cognitive performance [2]. The infections usually result from poor access to clean water, sanitation and hygiene. The most frequent STH infections are caused by roundworms *(Ascaris lumbricoides)*, whipworms *(Trichuris trichiura)* and hookworms (*Necator americanus and Ancylostoma duodenale*) [3]. Roundworms and whipworms occur when individuals accidentally ingest worm eggs from contaminated soil, food, drinking water or hands whilst infection with hookworm larvae occurs through skin contact with contaminated soil or ingestion of larvae [2].

The World Health Organisation (WHO) recommends periodic mass treatment for all children aged 1–15 years in areas in which STH prevalence exceeds 20% [4]. In Tanzania this policy is implemented through the National Neglected Tropical Disease Control Programme that conducts annual deworming campaigns in schools. In line with WHO recommendations, the programme uses a single dose of 400 mg albendazole for the treatment of STH infections [5]. Despite these efforts, surveys indicate that STH infections can be highly prevalent in parts of Tanzania [6], including in the Kagera region in the northwest of the country where the prevalence of infection with *A lumbricoides* and *T trichiura* may exceed 50% in primary school students [7]. Since deworming campaigns do not target the root causes of STH and re-infection occurs rapidly, an integrated approach that combines sustainable hygiene behaviour change with deworming could prove an effective way to control STH infections [8,9].

In this report, we describe the intervention design and the baseline survey results from an ongoing cluster-randomised controlled trial (c-RCT) conducted in 16 primary schools of the Kagera region in north western Tanzania. The trial aims to determine whether a hand hygiene intervention can sustain the effects of deworming on the prevalence of *A lumbricoides* and *T trichiura*.

## Materials and methods

### Study setting

Kagera region is a predominantly rural area of Tanzania situated on the western shores of Lake Victoria. The region comprises eight districts and has a total population of about 2.5 million [10]. The economy is mainly based on agriculture. About 90% of children attend primary school for at least some years [11]. Primary schools comprise classes 1 to 7 and may have between 500 and 1500 children, most of them aged 6–12 years. The average number of students per classroom in 2019 was about 80 (range 65 to 93) [12].

**Fig 1:**
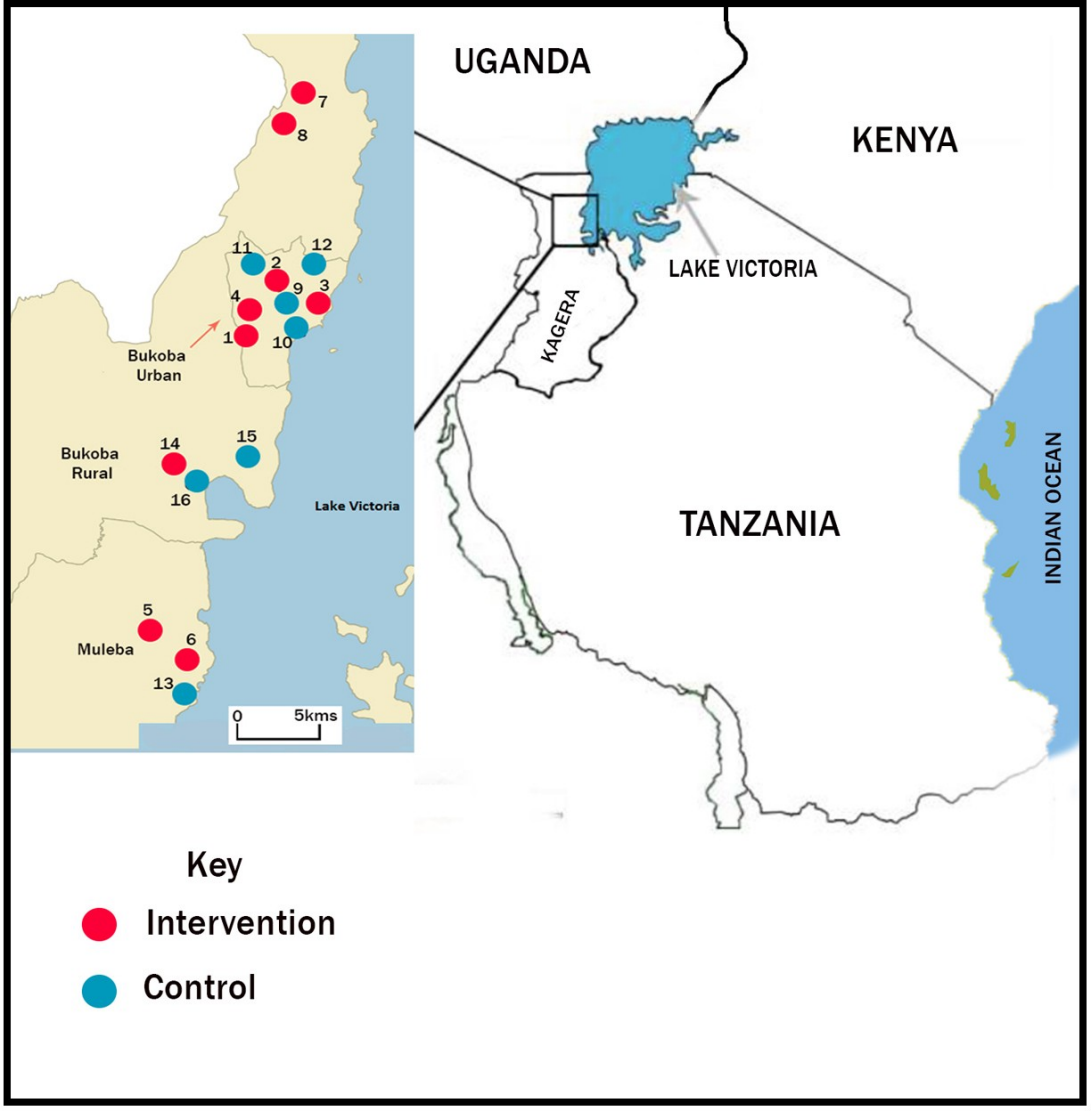
Map showing study sites in Kagera region, Tanzania.

**Fig 2:**
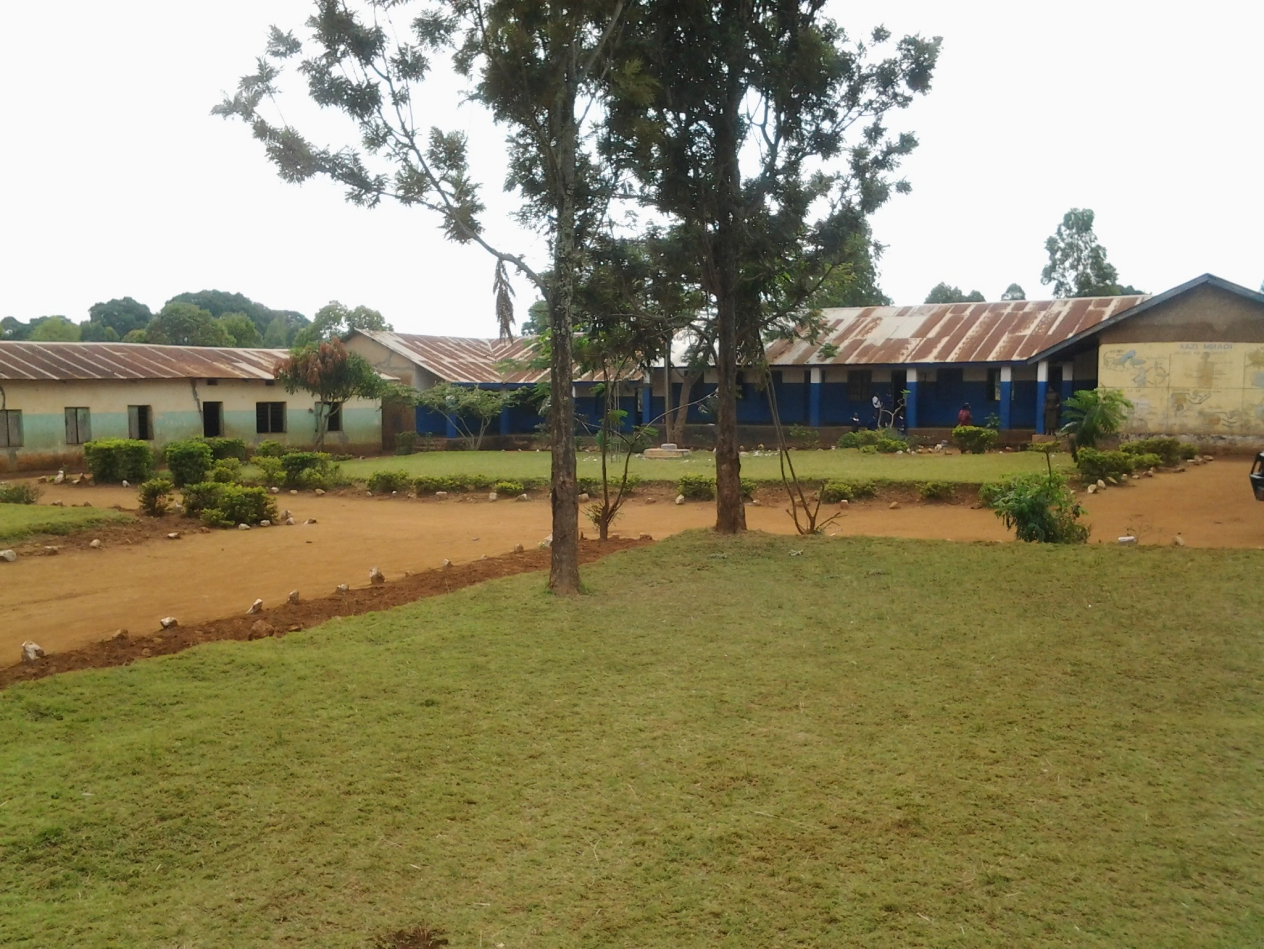
A picture of a primary school from Kagera region.

In 2017, we conducted an initial survey of STH prevalence in 51 schools across the three districts that border Lake Visctoria, i.e. Bukoba municipality, Bukoba rural and Muleba (**Fig 1**). From these, we recruited 16 primary schools for further participation in our study. Eligibility requirements were: a combined prevalence of ascariasis and trichuriasis of at least 20%; the total number of students not exceeding 1200; access to water within the school premises; and easy accessibility to the study team by road throughout the year. A typical environment of primary schools in Kagera region is shown in **Fig 2**.

The study is registered in the International Standard Randomised Controlled Trial Registry (ISRCTN45013173).

### Study design

This is an ongoing c-RCT, with primary schools randomised to the intervention (N = 8) or the control (N = 8) arm of the trial. Randomisation was stratified per district and restricted to ensure that schools with different levels of STH prevalence (as determined during the initial survey of 2017) were distributed equally across the study arms. There were 398 possible combinations that met this condition.

### Community participation and public randomization ceremony

To achieve maximum buy-in from the participating institutions, a meeting was held in Bukoba involving representatives of the 16 schools, the 3 district education officers and local administrators. During the meeting, the purpose of the trial and the planned intervention package was described, followed by a discussion with the audience. A computer-generated list of the 398 possible combinations, each comprising 8 schools in each trial arm, was presented. The final allocation of schools to their respective trial arm was performed by 3 representatives of the audience who drew numbered tennis balls from an opaque container. The sequence of the resulting digits indicated the chosen allocation on the list.

### Intervention development

Between September 2017 and April 2018 we conducted formative research to develop a combination intervention package and assess its feasibility and acceptability. Intervention design drew on experiences from studies in India [13], Kenya [14,15] and Bangladesh [16], and included a consultative process involving researchers and representatives from the regional and municipal education offices. The resulting package was piloted in 3 primary schools from Bukoba municipality, which were subsequently excluded from the main study. Experiences from the pilot phase were used for further adaptation of the intervention.

### Intervention components

**Fig 3:**
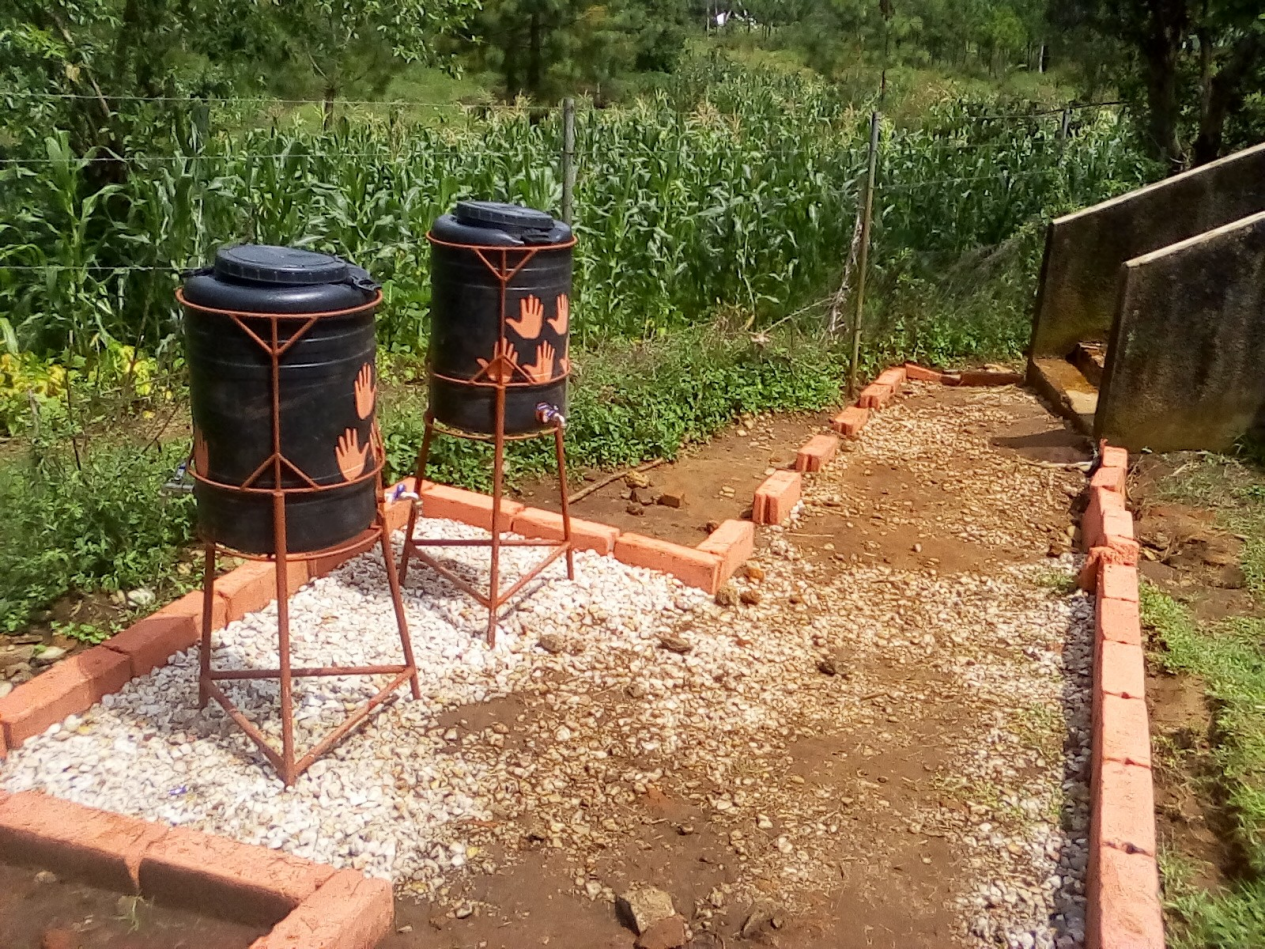
Handwashing facility with painted nudges from one intervention school.

**Fig 4:**
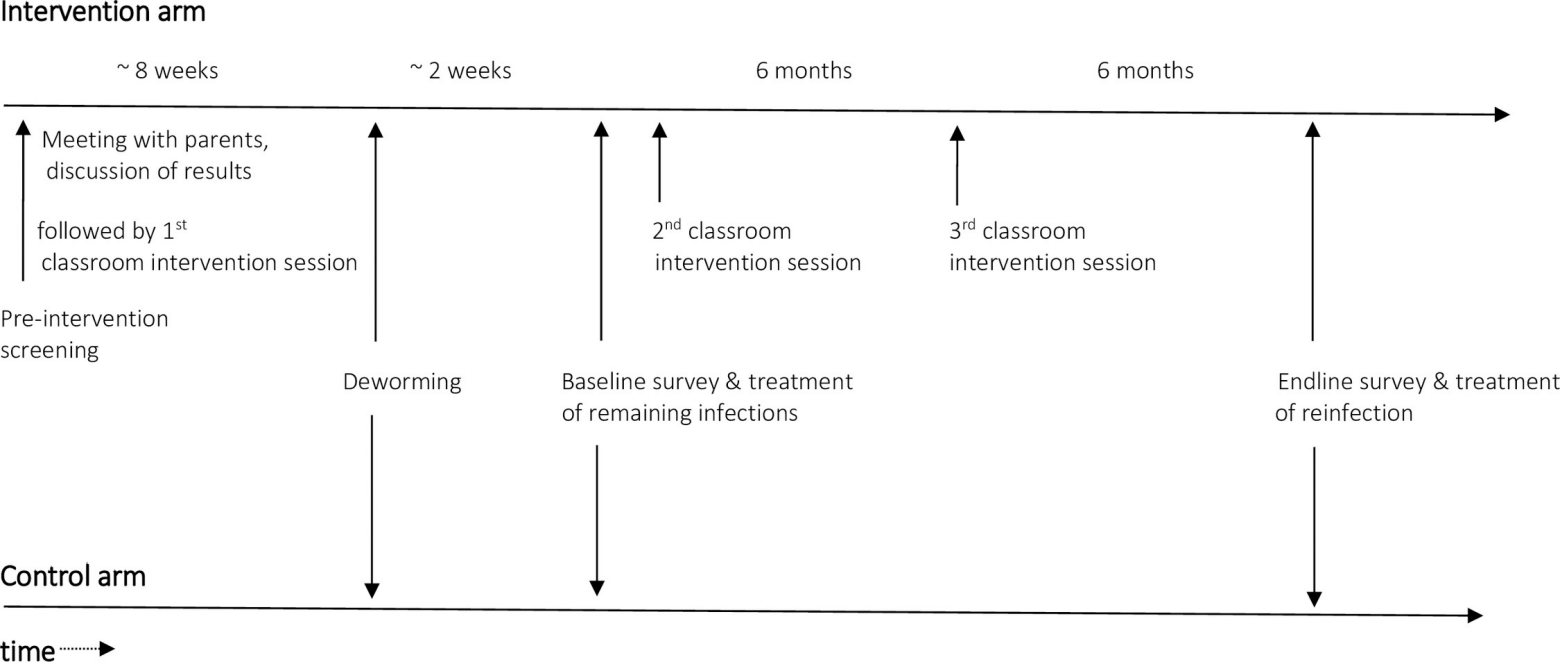
Relative timing of intervention events, surveys and deworming campaign.

The intervention combined water, sanitation and hygiene (WASH) related activities including improvement of water supply system at all intervention schools, and enhanced cleanliness of toilet facilities. The goal was to help children to regularly wash their hands after visiting the toilet and before eating. The final intervention has 3 complementary components:

- Parental engagement: Prior to study implementation, all students from intervention schools provided a stool specimen for STH infection screening. Subsequently, their parents were invited to attend a meeting at school where a project staff member gave a presentation about STH infections including causes, health consequences, and preventive measures with an emphasis on hand washing hygiene at key times. A short information leaflet was also distributed (**S1 and S2 Appendices**). Parents were then individually provided with the stool test results of their children, issued in closed envelopes. Prevention of STH infections was discussed in detail and the importance of washing hands at key times reiterated. Parents were also prompted to consider options for improving hand washing practices at home. The principle underlying this parental engagement strategy was to create personal emotional concern among parents to support behaviour change efforts among their children and the entire family.
- Structural changes within the school environment: A range of simple measures were implemented in the intervention schools to encourage hand washing with water and soap. First, about 4–6 improved locally produced handwashing stands were placed near the school toilets (which typically consisted of cubicles with pit latrines) in each school (**Fig 3**). Each stand consisted of a 100-litre plastic water tank with two taps. Schools established duty rosters for students to check and refill tanks and supply soap regularly, under the supervision of a dedicated teacher. In addition, footpaths were constructed connecting the handwashing station to the toilet block and were marked with brightly coloured bricks. The same colour was used to paint handprints on the handwashing stands (**Fig 3**). These colour marks served as environmental nudges to prompt hand washing behaviour, like those used successfully in a school health project in Bangladesh [16].
- Classroom-based hygiene promotion: Three teachers were trained in each intervention school to conduct 3 sessions over the course of 1 year, each lasting about 60–80 minutes. The intervention started with a kick-off session at the beginning of the trial just before the annual deworming campaign. Schools held two additional “booster sessions” after approximately 6 and 12 months (**Fig 4**). Sessions were held in all classes of standard 1 to 6. The initial sessions were conducted in collaboration with the project intervention team. Teaching content was based on an approved amendment to the existing school curriculum as this did not yet include a section on hand hygiene (**S3 and S4 Appendices**). General information on STH infections was included, adapted to different age groups. Lessons focused on important emotional drivers that would motivate participants to adopt better hygiene behaviour. Teaching used materials developed during the formative research phase, including a play and a demonstration of correct hand washing procedures. Hygiene promotion messages centered on two primary characters–a school aged boy and girl that were repeated in various stories, posters, and images.

### Intervention measures provided in both trial arms

- Maintenance of existing water supply systems: All 16 schools participating in the project had access to water within their premises. Most schools had a rainwater harvesting cistern with taps, some were linked to a piped water system. In many schools the infrastructure required small-scale repairs which the project facilitated.
- Deworming campaigns: Routine annual deworming campaigns which involve supervised oral medication with 400 mg albendazole given to all children in primary school had been conducted by the National Neglected Tropical Disease Control Programme about 10 months prior to the start of the trial. In agreement with the Ministry of Health, the study team implemented the subsequent campaign on behalf of the national programme, using identical medication and dosage, in collaboration with health workers from the Regional Medical Office of Kagera region.

Deworming was completed just after all intervention components had been put in place in intervention schools (**Fig 4**). The rationale for this sequence of events is that infections found at the endline survey are more likely to be reinfections that occurred in spite of the intervention, and differences between infection prevalences in the two arms may be attributed to the intervention.

### Impact evaluation

The evaluation comprises a baseline survey conducted just after the deworming campaign in both arms of the trial, and just after the intervention has been started in the intervention arm; and an endline survey to be conducted about 12 months after the baseline survey in each school (**Fig 4**).

### Primary outcome

This is defined as the combined prevalence of ascariasis and trichuriasis to be determined at the time of the endline survey, conducted after the intervention had been in place in intervention schools for about 12 months (**Fig 4**). The presence of STH infection is determined microscopically.

### Secondary outcomes

These include:

- hand-washing behaviour in schools (reported and observed) and at home (reported only), 12 months after deworming
- intensity of ascariasis and trichuriasis infections 12 months after deworming
- prevalence and intensity of hookworm infection 12 months after deworming
- levels of hand contamination with STH eggs and E. coli bacteria 12 months after deworming

### Baseline survey and STH screening

The baseline survey had three objectives: (i) to confirm whether the deworming campaign was successful, and identify and treat students from both arms who are still infected, (ii) to document any baseline differences in STH prevalence and other relevant covariates between trial arms in order to facilitate the analysis and interpretation of trial results, and (iii) measure reported handwashing behaviour and water, sanitation, and hygiene access at school and home.

From each of the 16 participating schools we aimed to recruit 200 students, stratified by class. A random selection was made of 35 students per class, stratified to ensure equal representation of boys and girls. Assenting students were interviewed using a structured questionnaire (**S5–S7 Appendices**) to obtain information about demographic characteristics, availability of water at home, soil ingestion, food preservation and reuse, and current handwashing practices. Students were also asked if they had ever observed excreting worms during defecation. The interview took about 30 minutes per student, pauses were made for younger students, or if a student seemed tired. Participants were issued stool containers and spatula and were asked to provide a stool specimen just before or immediately after the interviews. Students who were unable to provide stool samples on the day of interviews met with research team to schedule another time within the same week to provide the samples.

### Endline survey

The endline survey has the objective to collect data on the primary and secondary outcomes. It will follow the same procedures as the baseline survey and will be conducted about 12 months after the deworming.

At the end of the study, we will also conduct a sub-study on hand contamination (**S8 and S9 Appendices**), using hand-rinse water which will be collected from 20% of the students who participate in the endline survey (about 640 children in total), and will be examined for the presence and concentration of STH ova and for the presence and concentration of *Escherichia coli bacteria*. A short questionnaire will be administered to collect demographic information, last time the student went to the toilet to defecate, last time hands were washed with/without soap, and also on activities performed during the day that may have led to the contamination of hands with faecal material. This sub-study will help to obtain additional evidence to explain the results of the main trial.

### Laboratory procedures

Stool samples collected for pre-intervention STH screening in the intervention schools were tested using the Kato-Katz technique [17]. This technique was used because it is simple and allows same day processing of a large number of samples collected in the field. The purpose of this activity was to give feedback to parents and engage them in the discussions of prevention strategies. During the baseline survey, stool samples from both intervention and control schools were analysed using the formol-ether concentration technique to identify helminth ova and protozoa cysts, and to determine infection intensity [17,18]. For safety reasons, ether has been replaced by ether-acetate, which has been shown not to affect the results [19]. Each slide was read by two independent technicians and when results were different a third reading using freshly prepared slide was done by another technician. The same methods will be applied to stool samples collected during the endline survey.

For the sub-study on hand contamination, the number of ova on hands will be assessed through a concentration technique using McMaster slides [20]. A 100 ml sample from the hand rinse wash water will be used for the detection of STH ova and of *Escherichia coli bacteria*.

### Sample size calculation

The study was powered to show a 40% to 50% relative reduction in combined prevalence of ascariasis and trichuriasis infection between the intervention and control arms at the endline survey. We hypothesised that in the absence of a handwashing intervention following the deworming campaign, the prevalence of helminth infection in the control arm would reach original levels of 30%, reported in pilot study, within one year. Assuming a prevalence of 30% for combined helminth infection in the control arm and a between cluster coefficient of variation (k) of 0.3 based on pilot data, and a total of 16 primary schools in the trial with 200 participants per school (total of 3200 participants across schools), we determined that the trial would provide at least 80% and 95% power for intervention effects of 40% and 50%, respectively.

### Statistical analysis of baseline data

No formal statistical hypothesis testing was planned for the baseline data, consistent with recent guidelines for statistical reporting of study results [21]. Therefore, a descriptive analysis was conducted. The demographic and household characteristics of participants was tabulated by intervention arm. Data on water, sanitation and hygiene were similarly analysed using frequency tabulations to describe household access to clean and safe water, and sanitation facilities. The prevalence of worm infection was estimated by calculating the percentage of children in each trial arm with stool samples that were positive for STH ova. Infection with either ascaris and/ or trichuris was calculated followed by separate calculations for prevalence of infection with each STH and with hookworm. The prevalence of combined STH infection was presented for both trial arms using frequency tabulation. Similar descriptive analysis was repeated for each of the secondary measurements of ascariasis, trichuriasis and hookworm prevalence.

### Ethical considerations

Separate ethical approval was obtained for the formative research and for the main trial from the ethics committees of the Tanzanian National Health Research Ethics Committee of the National Institute for Medical Research (Ref: NIMR/HQ/R.8a/Vol. IX/2321 and 2497), and the London School of Hygiene and Tropical Medicine (Ref: LSHTM 11810 and 11868). All students in the intervention arm are exposed to the intervention. Because health education is a principal part of the existing school curriculum at primary schools in Tanzania, and because hygiene promotion and handwashing after defecation and before meals are not invasive procedures, ethics committees agreed that consent or assent was not required for the intervention. The same applies to deworming campaigns that are routinely conducted at primary schools.

In order to enrol students for the baseline survey, students were asked to provide informed written assent. Students who were not yet able to read and write provided verbal assent which was confirmed in writing by a witnessing teacher. Parents or guardians were invited to provide informed consent based on a circulated information leaflet, using an opt-out strategy for those who did not wish their children to participate. The same procedures will be applied at the endline survey.

## Results

### Phased roll-out of study procedures

The study began in November 2017. Sixteen primary schools are included, of which 8 have been allocated to the intervention arm and 8 to the control arm of the trial. Half of the schools are in Bukoba municipality and half in predominantly rural districts (5 in Bukoba rural and 3 in Muleba). The intervention was phased in stepwise at intervention schools between November 2017 and June 2018. Deworming of the entire school population was conducted two weeks after the intervention was in place at intervention schools and simultaneously in corresponding control schools within the same district.

### Baseline survey and STH screening

At each school, the baseline survey was conducted about 2 weeks after deworming (**Fig 4**). Out of 3,360 students we randomly sampled from 16 schools, 3,281 (97.6%) consented to take part in the baseline survey. Of these, 3,163 (96.4%) were interviewed (1582 in the 8 intervention schools and 1581 in the 8 control schools) and 3,131 (95.4%) students provided stool samples. Students were not interviewed or did not provide stool samples mainly because they were absent at the time of enrolment. Baseline activities took about 1 week in each school. The endline survey will be equally phased in and is scheduled to begin about 12 months after the baseline survey was performed in each school.

### Demographic data

By design, about half of the enrolled participants were girls (1616, 51%), and equal numbers of students were recruited from each class. The age ranged from 6 to 14 years, with a median of 10 years (**Table 1**). About 68% of these students lived in households with both their parents, 16% with one parent and another 16% were looked after by a guardian.

**Table 1:** Characteristics of primary school students during the baseline survey of *Mikono Safi* trial, Kagera region, Tanzania.

	Overall N (%)	Intervention N (%)	Control N (%)
**Total sample**	3163	1582	1581
**Age in years**			
Median [interquartile range, IQR]	10[8 to 12]	10[8 to 12]	10[8 to 12]
**Sex**			
Male	1547 (49)	775 (49)	772 (49)
Female	1616 (51)	807(51)	809 (51)
**Participants currently living with**			
Both parents	2150 (68)	1069 (68)	1081(68)
Single parent	494 (16)	251 (16)	243 (16)
Non-biological parents	519 (16)	262 (16)	257 (16)
**Sources of households’ drinking water**			
In-house piped water	448 (14)	166 (10)	282 (18)
Public owned piped water	503 (16)	239 (15)	264 (17)
Well	216 (7)	99 (6)	117 (7)
River / stream	1960 (62)	1040 (66)	920 (58)
Lake	61 (2)	51 (3)	10 (1)
Water vendor	79 (3)	41 (3)	38 (2)
Other sources	82 (3)	45 (3)	37 (2)
Unknown	3 (0)	2 (0)	1 (0)
**Drinking water treatment practices at households**			
Filtering with a cloth	857 (27)	418 (26)	439 (28)
Ceramic filter/candle	4 (0.1)	1 (0)	3 (0)
Boiling	2077 (66)	1023 (65)	1054 (67)
Chemical treatment	51 (2)	18 (1)	33 (2)
Use presumed safe piped water	166 (5)	77 (5)	89 (6)
Other practices	553 (17)	306 (19)	247 (16)
Unknown	68 (2)	34 (2)	34 (2)
**How cooked food is preserved at households**			
Left uncovered	17 (1)	10 (1)	7 (0)
Kept in a covered container	2966 (94)	1456 (92)	1510 (96)
Other practices	106 (3)	67 (4)	39 (2)
Unknown	74 (2)	49 (3)	25 (2)
**How left-over food is prepared for re-use at households**			
Nothing done	385 (12)	181 (11)	204 (13)
Heating or boiling	1737 (55)	919 (58)	818 (52)
warming	1016 (32)	464 (29)	552 (35)
Other practices	38 (1)	9 (1)	29 (2)
Unknown	65 (2)	35 (2)	30 (2)

### Water sources at household level

Most students (62%) reported that a river or stream was the main drinking water source at home. About 30% had access to piped water, either within their community (16%) or in the house (14%). Almost all of these resided in town. Some households (7%) obtained water from a well. According to children’s reports, nearly all households treated their water before drinking, mostly by boiling (66%) or filtering it through a piece of cloth (27%) (**Table 1**).

### Food preservation and reuse

Nearly all children (94%) reported that at home, freshly prepared food would be kept in a covered container until use. However, 12% said that left-over food would be used again without boiling or intensive reheating.

### Handwashing practices

Almost all students (97%) reported that they had washed their hands at least once on the day that preceded the interview; and most reported to have washed them 2 or 3 times. At the time of the interview, 66% of the students had washed their hands on that day so far. When asked on which occasions they would usually wash their hands, 76% responded to do so before eating, and 53% said they do so after visiting the toilet. When students were asked about the reasons for washing hands the last time they had done so, 48% responded to do so before eating, and 54% said they did so after visiting the toilet (**Table 2)**.

**Table 2:** Description of sanitation facilities and hygienic practices reported by primary school students during the baseline survey of *Mikono Safi* trial, Kagera region, Tanzania.

	Overall N (%)	Intervention N (%)	Control N (%)
**Total sample**	3163	1582	1581
**Hand washing history**			
Students washed hands on interview day	2093 (66)	1117 (71)	976 (62)
Number of times washed hands, Median [IQR]	1 [1–2]	1 [1–2]	1 [1–2]
Students washed hands on day preceding interview	3058 (97)	1523 (96)	1535 (97)
Number of times washed hands, Median [IQR]	2 [2–3]	2 [2–3]	2 [2–3]
**When do you usually wash your hands?**			
Before eating	2419 (76)	1122 (71)	1297 (82)
After visiting the toilet	1703 (53)	1009 (64)	694 (44)
Other times	714 (23)	312 (20)	402 (25)
Don’t know / don’t remember	82 (3)	53 (3)	29 (2)
**Recently there was an occasion when student was unable to wash hands although intended to do so**	968 (31)	518 (33)	450 (28)
Reasons preventing students to wash hands during the last time they intended to do so*			
Was in a hurry	107 (11)	36 (7)	71 (16)
I forgot	200 (21)	94 (18)	106 (24)
There was no water	311 (32)	199 (39)	112 (25)
There was no soap	83 (9)	59 (11)	24 (5)
I don’t know	41 (4)	15 (1)	26 (2)
Other	226 (23)	115 (22)	111 (25)
**What were the reasons which made students to wash their hands the last time they did so?**			
Had visited the toilet	1702 (54)	1119 (71)	583 (37)
Washed before eating	1526 (48)	608 (38)	918 (58)
Was told to wash hands	60 (2)	24 (2)	36 (2)
Hands were dirty	280 (9)	110 (7)	170 (11)
Don’t remember	28 (1)	16 (1)	12 (1)
Other reasons	365 (12)	125 (8)	240 (15)
**Materials used by students to wash their hands the last time they did so**			
Water only	1032 (33)	328 (21)	704 (45)
Water and soap	2125 (67)	1254 (79)	871 (55)
Other materials	3 (0)	0 (0)	3 (0)
Could not recall	3 (0)	0 (0)	3 (0)
**Students reported having a latrine at home**	3156 (99.8)	1577 (99.7)	1579 (99.9)
Type of latrine available at home**			
Flush latrine	1229 (39)	591 (37)	638 (40)
Pit latrine	2018 (64)	971 (62)	1047 (66)
Pit latrine with cover lid	842 (27)	407 (26)	435 (28)
Latrine has a ventilation pipe	1041 (33)	515 (33)	526 (33)
Materials used to construct toilet at home**			
Brick walls	1470 (47)	684 (43)	786 (50)
Metal roof	1986 (63)	957 (61)	1029 (65)
Cement floor	1392 (44)	673 (43)	719 (46)
**Materials used for anal cleaning the last time passed stool**			
Water	2100 (66)	997 (63)	1103 (70)
Leaves/plant materials	533 (17)	307 (19)	226 (14)
Garbage paper	230 (7)	127 (8)	103 (7)
Toilet paper	202 (6)	78 (5)	124 (8)
Other	152 (5)	93 (6)	59 (4)
Nothing	2 (0.1)	0 (0)	2 (0.1)
Could not recall	4 (0.1)	2 (0.1)	2 (0.1)
**Reported ever eaten soil**	1098 (35)	523 (33)	575 (36)
**History of diarrhoea**			
Had ≥ 1 episode of diarrhoea over the past 7 days	1040 (33)	512 (32)	528 (33)
Had ever observed worms while passing stool	1600 (51)	783 (49)	817 (52)

*This was among students who reported an occasion when they wanted to wash hands but could not do so.

**This information was obtained only from students reporting to have a latrine at home.

The majority of students (67%) reported that when they washed hands, they would use both water and soap whilst the remainder would use water only. About one third (31%) said that occasionally they did not wash hands although they wanted to. Several reasons were given of which the lack of water was the most frequent one mentioned (**Table 2**).

### Toilet facilities at home

Nearly all households had access to a toilet, mostly a pit latrine (64%). About 40% of students reported that their toilet was operated with flush water, almost all of these lived in town. Toilets typically had a concrete or wooden floor, a wall made of bricks or clay, and a roof comprising corrugated iron sheets. A minority of latrines were equipped with a ventilation pipe for smell and insect control (33%) (**Table 2**).

### Other behaviours potentially associated with intestinal infections

In Kagera as elsewhere in Tanzania, commercially purchased toilet paper is not routinely available at toilets, neither at school nor in the home. Most students reported that for anal cleaning after defecation, they apply water (66%), whilst 17% stated that they use plant material, waste paper (7%), or other materials (5%). Only six percent said they used toilet paper (**Table 2**).

We specifically asked about soil eating habits as we were aware from other studies in Africa that geophagy may frequently occur among primary school children in some areas [22]. About one third of our participants (35%) confirmed that they have sometimes practiced this (**Table 2**).

There were no important imbalances between trial arms with regards to demographic characteristics, sources of drinking water and water treatment practices at home (**Table 1**). Practices used to preserve freshly prepared food were also similar, but slightly more students in the intervention than in the control arm reported that left-over food would be heated or boiled before further use (58% vs. 52%). We did not observe important differences with respect to the availability or type of toilets at home (**Table 1**), the type of material used for anal cleaning after defecation (**Table 2**), reports of recent diarrhoea and reported excretion of worms in the past (**Table 2**).

Reported hand washing on the day prior to the baseline survey interview did not differ significantly. However, more children in the intervention arm reported handwashing at the day of the interview (71% vs 62%), handwashing after using the toilet in general (64% vs. 44%) or the last time they had washed their hands (71% vs. 37%). On the other hand, children in the control arm reported better handwashing behaviour than their school mates in the intervention arm with regards to handwashing before eating, both in general and for the last time they washed hands (82% vs 71%; and 58% vs 38%, respectively). More students in the intervention arm reported using soap when handwashing (79% vs 55%) (**Table 2**).

### Reported intestinal infections

Fifty-one percent of students had ever observed excreting worms during defecation. The interview question did not further specify which kind of helminth was discharged nor whether this had occurred spontaneously or after deworming.

One third of the students had experienced an episode of diarrhoea during the week before the interview (**Table 2**).

### STH prevalence following deworming

Stool specimens were available from 3131 participants, collected at the time of the baseline survey about 2 weeks after deworming (1570 from the intervention schools and 1561 from the control schools).

Overall, ova of *Ascaris lumbricoides* were still found in 55 students (1.8%) and of *Trichuris trichiura* in 1072 students (35%). In total, 1095 participants (35%) had either or both infections (**Table 3**). All students who had STH infection after deworming had low intensity infections.

**Table 3:** Prevalence of soil transmitted helminth infections after deworming among primary school students in *Mikono Safi* trial, Kagera region, Tanzania.

	Overall	Intervention	Control	OR (95% CI)	P value
**Total sample**	**3131**	**1570**	**1561**		
Ascaris					
Prevalence, n (%)	55 (1.8)	6 (0.4)	49 (3.1)		0.051
*Arithmetic mean egg count (SD)	191.6 (396.1)	79 (57.4)	205.4 (417.6)		
Trichuriasis					
Prevalence, n (%)	1072 (34)	543 (35)	529 (34)		0.962
*Arithmetic mean egg count (SD)	19.1 (53.6)	20.6 (58.4)	17.6 (48.0)		
Ascariasis and/or trichuriasis					
Prevalence, n (%)	1095 (35)	543 (35)	552 (35)		0.885
Hookworm					
Prevalence, n (%)-	19 (0.61)	7 (0.45)	12 (0.77)		0.309
*Arithmetic mean egg count (SD)	7 (13.7)	12.1(21.6)	4 (4.9)		

* Measured as eggs per gram.

The prevalence of ascariasis after deworming was somewhat higher in the control arm than in the intervention arm (3.1% vs. 0.4%). The prevalence of trichuriasis after deworming was equally high in both arms, and this was also the case for both infections combined (**Table 3**).

## Discussion

### Intervention and study design

We presented the design and the baseline findings of a school-based intervention to promote handwashing with water and soap at key times to reduce the prevalence of STHs, given that mass STH treatment alone is not effective in preventing reinfection. Only few studies have addressed this important issue, with some studies showing this to be effective [23,24], while other studies showing it to be ineffective [25,26], or only partially effective [14]. These inconsistent results are in part related to the intervention strategy used [25]. The intervention we developed in this study is innovative as it combines classroom-based hygiene promotion in schools, creation of personal emotional concern among parents and guardians, and applying nudges to trigger improved hand-washing behaviour. Emotional drivers aimed to generate a feeling of disgust by making parents and children understand that ascariasis and trichuriasis are caused by ingesting faecal matter. This was reinforced by sharing children’s stool test results with their own parents after explaining transmission routes. Nudges to encourage handwashing with soap have been successfully applied in two previous trials but have not been examined with respect to their effect on STH infection [27,28].

Our study is a c-RCT. This design is required when interventions are addressed to entire groups rather than individuals and when ‘herd immunity’ effects are desired, such as the influence of peers [29]. These types of trials are usually open label where allocation concealment can only be maintained during randomisation and data analysis. In addition, blinding was not possible in this trial due to the nature of the intervention tested.

The design of our study is unusual in two regards: firstly, we use two subsequent cross-sectional studies rather than a cohort design in order to evaluate intervention effects. This approach has been chosen for two reasons: Firstly, mobility and non-attendance among primary school students in Kagera (as in some other parts of Africa) can be high and sufficiently high cohort follow-up rates may be difficult to achieve. Furthermore, the intervention is being provided to all children in the participating intervention schools and not just to the sub-group selected for the evaluation, and therefore an intervention effect should be demonstrable in any subgroup, as long as the group is randomly chosen.

Secondly, as shown in Fig 4, the intervention was launched a few weeks before the baseline survey. This sequence of events followed a reverse order compared to that commonly applied in intervention trials. We chose this approach to avoid that during the two weeks between the deworming campaign in all schools and the baseline survey, rapid reinfection might occur in the intervention arm because the intervention was not yet in place.

### Baseline survey and STH screening results

Our results show that the study is being conducted in a population of primary school students typical for rural communities and small towns in East Africa. WASH facilities are available but are of modest quality. STH prevalence can be high under these conditions as evidenced by our results.

The high prevalence of STHs in certain areas of Tanzania has been described by others [7]. Kagera region is one of these areas which made it an ideal environment for this study to examine the effect of handwashing on STHs. Some other areas in Tanzania are marked by low STH prevalence, and it is likely that this is associated with local climate and soil conditions rather than population differences in WASH behaviours [30].

Open defecation is common in many parts of rural Tanzania [31] as elsewhere in sub-Saharan Africa, and so it is somewhat surprising that nearly all children in our study reported to have access to a toilet at home, mostly a pit latrine. A qualitative household-based study currently underway in the project area seems to confirm children’s reports.

### Comparison of baseline characteristics between trial arms

In general, the randomisation worked well in our study: demographic factors including access to water and sanitation facilities were equally distributed between trial arms at baseline. We did also not observe important differences with respect to the availability or type of toilets at home, the type of material used for anal cleaning after defecation or reported geophagy.

There was some imbalance between baseline data across trial arms with regard to self-reported handwashing behaviour. The direction of this was inconsistent: the prevalence of reported handwashing after defecation was higher in intervention schools than in comparison schools, and the same applied to the use of soap; in contrast, handwashing before eating was more frequently reported by children in control schools. A possible explanation for the observed imbalance in reported post-defecation washing practice could be that at the time of the baseline survey the intervention had already just been launched in intervention schools for the reasons described above; but one would then expect a similar trend for handwashing before meals which we did not observe. It is difficult to estimate the net effects of the contrasting imbalances.

We did not find substantial differences in the distribution of STHs, neither for the combined prevalence of trichuriasis and ascariasis, nor for trichuriasis alone. However, there was a difference in ascariasis favouring the intervention arm: the prevalence was low in both trial arms, but about seven times higher in control than in intervention schools (Table 3). We have no explanation for this difference and expect it to be a chance finding. Importantly, all children still found infected at baseline were treated once again, so that we would expect a reduction or resolution of the observed imbalance.

The main strength of our study is its cluster-randomised design that allows to document the potential effect of the intervention on handwashing practices. A further positive aspect is the expected strengthening of improved hygiene behaviour as a result of parental engagement, and because of the use of nudges to encourage handwashing with soap. These components employ emotional and partially subconsciously motivating elements that are expected to go far beyond the effects of mere knowledge transfer that can be achieved through verbally performed classroom-based health education sessions.

In c-RCTs, the baseline variation in the prevalence of the outcome variable is often not known, and so the inter-cluster coefficient of variation is difficult to predict, potentially resulting in either an insufficient or a higher than necessary number of clusters. Fortunately, at the planning stage of our trial we studied (unpublished) infection prevalence data from more than 50 primary schools in Kagera region. This allowed us to determine the inter-cluster coefficient beforehand.

To determine STH prevalence at baseline and endline surveys, we used the formol-ether concentration technique which has consistently shown higher recovery, especially in light infections, in comparison to other techniques including the frequently applied Kato-Katz technique [17,18].

The trial also has some limitations. Trial outcomes include hand washing behaviour and hygiene practices based on student reports and may be subject to desirability bias which may even differ between trial arms. A matter of concern is the high prevalence of trichuriasis in spite of deworming. It is possible that the study may show a strong effect on ascariasis prevalence and intensity, but a smaller effect on trichuriasis. Because the primary outcome of the trial is defined as the combined prevalence of ascaris and trichuris infection, it is possible that the study may not detect a significant effect of the intervention on this outcome. However, if the intervention were truly effective in improving handwashing practice and reducing the prevalence of STHs in principle, we would still expect major effects on the secondary outcomes of the study.

Our observation suggests that the treatment currently used in the national deworming campaigns (single dose of 400 mg albendazole) may be insufficient to cure *Trichuris trichuria* infection. We have communicated our findings to the Ministry of Health at national and regional levels. The limited effects of this treatment regimen on trichuriasis has been reported also by others [32]. This could be improved if albendazole were given in multiple doses or combined with other antihelminthics, e.g. ivermectin or mebendazole [33–35].

### Conclusion

We described the design and baseline observations from an ongoing c-RCT conducted at primary schools in Kagera region, Tanzania. The study is being conducted in the context of regular school-based annual deworming campaigns and aims to determine the effectiveness of an innovative combination intervention in inducing handwashing with water and soap at key times and reducing STH prevalence. Baseline survey results show a generally well-balanced distribution of potential confounding factors across trial arms. Baseline survey results suggest that routine deworming with 400 mg albendazole is not effective in treating *Trichuris trichiura* infection. The trial is expected to report in early 2020.

### Roles of coinvestigators

The trial was designed by JE, HG, CH, KM, Ski and Ska. Sample size estimates and randomization procedures were guided by CH. Formative research was planned by RD with input from KM, Ska and HG, and was implemented by RD, SR, EO and OM. The trial is being coordinated by KM with input from HG, PA, Ski and Ska. Baseline survey activities were coordinated by SS. Laboratory work was planned and supervised by Ski. Analysis of baseline survey data was performed by PA. KM, HG and Ska drafted the manuscript. All co-authors contributed critically to the manuscript and approved the final version.

## Supporting information

S1 Appendix Parent information leaflet–English version. (PDF)

S2 Appendix Parent information leaflet–Swahili version. (PDF)

S3 Appendix *Mikono Safi* training curriculum–English version. (PDF)

S4 Appendix *Mikono Safi* training curriculum–Swahili version. (PDF)

S5 Appendix Impact evaluation questionnaire–English version. (PDF)

S6 Appendix Impact evaluation questionnaire–Swahili version. (PDF)

S7 Appendix School level questionnaire–English version. (PDF)

S8 Appendix Sub-study questionnaire–English version. (PDF)

S9 Appendix Sub-study questionnaire–Swahili version. (PDF)
