# Supplementary material for: Hand hygiene intervention to optimize helminth infection control: Design and baseline results of Mikono Safi–An ongoing school-based cluster-randomised controlled trial in NW Tanzania
Source: PLoS One. 2020 Dec 9;15(12):e0242240. doi: 10.1371/journal.pone.0242240 (PMC7725373; doi:10.1371/journal.pone.0242240)

MIKONO SAFI TRIAL, KAGERA REGION

INFORMATION SHEET FOR PARENTS

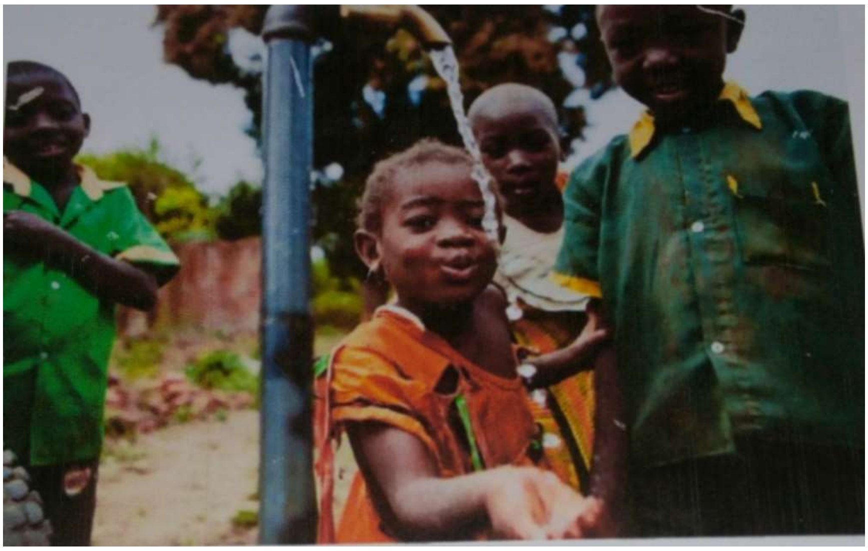

Why are we here?

- We are working together with the education department of Bukoba Municipal Council to improve handwashing among primary school children in your community. This will help protect your children from intestinal worms and improve health.
- Together with the Bukoba Municipal Council, we tested all children in standard one for intestinal worms. You will find your child's results in a separate envelope.

Why is it bad if your child has worms?

- If your child has worms, the worms can suck their blood and your child will have less blood than they are supposed to have.
- Worms compete for your child's food in their intestines, so your child can become malnourished.
- Having worms can make children tired a lot, which can make them less productive at school and at home.
- When a child has a lot of worms, they might need stomach surgery.

How does a child get worms?

- There are many ways in which children can get infected with worms. For example, your child can get worms if they use the latrine and don't wash their hands after or if they eat without washing their hands first.
- Some worms hatch in the soil and then burrow in the skin. If your children walk barefoot in the soil, they can get infected.
- Worm eggs can live in dirt, on fruit, or on someone's hands if they do not wash them after going to the latrine. If your child plays in the dirt, eats unwashed fruit, or touches dirty hands and then they insert their fingers in their mouth, they can get worms.
- Just because you do not see the worm eggs does not mean that they are not there. They are too small to see without using a special tool such as a microscope.

What do my child's test results mean?

**HAVE INFECTION**
**NO INFECTION**

- The attached sheet shows the type of common worm infections and the number of worm eggs per gram of stool we analysed. These results and numbers can be difficult to understand. To make this simpler, we have provided a color coding system. You will see that there are four types of worms to be tested and for each worm results will be shown using the background which is either green or red. For each type of worm tested, if your child has no infection, the background will be green. If your child has worm infection, the background will be red. If your child has infection, within the box with red background colour, there will be a number indicating the number of worm eggs per gram of stool we analysed. Higher number indicates much more severe infections. If the results show no infection for a particular worm, there will be no number in green boxes because there is no infection detected.

- For example: Results below shows that there are no infections for the first and third columns (green background colour), and there are infections for the second and fourth columns (red background colour). The level of infection is higher in fourth column than in second column.

S. mansoni	Hookworm	Ascaris	Trichuris
	890		6700

What do I do if my child has worms?

- As part of this project, we are giving medicine to every child that has worms. This is at no cost to you.
- However, there are steps you can take at home to help protest your child from worms in the future.

What can I do at home to keep my children from getting worms?

- Make sure that you can wash hands at home
    - Keep a a bucket of water, soap, and a jug to wash hands near the latrine.
    - Make sure supplies are near the latrine so your child sees them on their way out.
- Encourage your children to wash their hands
    - Talk to your child about handwashing. Ask them to show you how they wash hands.
    - Every time you see your child go to the latrine, remind them to wash their hands.
    - Every time your child is going to eat, even if it is a snack, make sure they wash their hands.
    - Discuss handwashing with your children and with other people in your households
- Always wash fruits with water before eating.
- Use water from a safe source. Boil or treat your water in the home to ensure it is safe. Even water that looks clean can have worms.

- Set a good example! Children learn by watching what others do. Being a good role model for children will encourage them to wash hands.

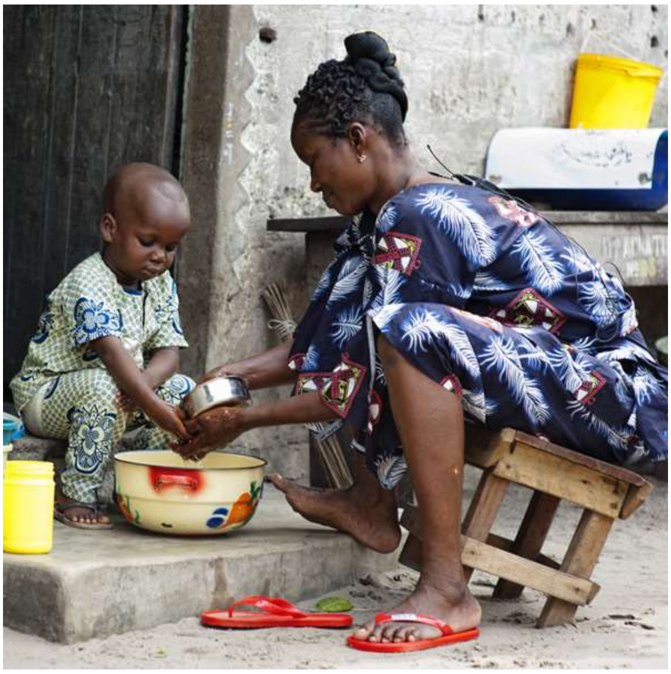

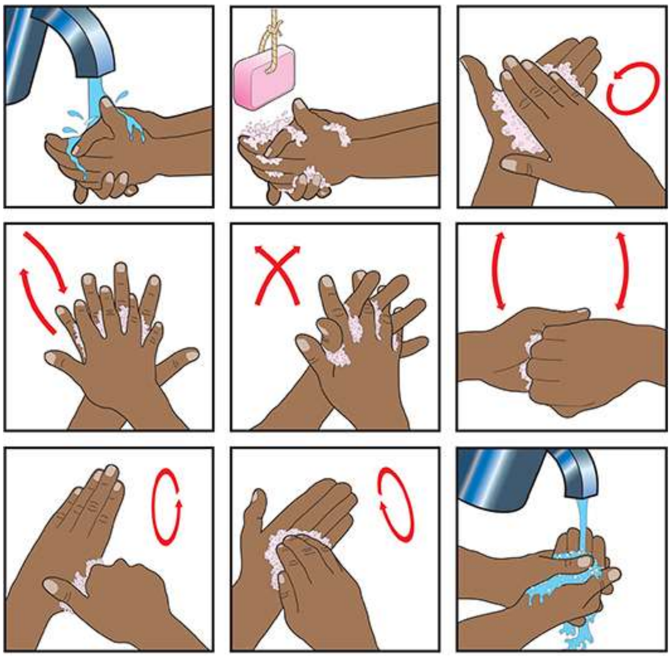

Supplement: S1 Appendix — (PDF) [file pone.0242240.s001.pdf]
