# Supplementary material for: Hand hygiene intervention to optimize helminth infection control: Design and baseline results of Mikono Safi–An ongoing school-based cluster-randomised controlled trial in NW Tanzania
Source: PLoS One. 2020 Dec 9;15(12):e0242240. doi: 10.1371/journal.pone.0242240 (PMC7725373; doi:10.1371/journal.pone.0242240)

MPANGO WA UTAFITI WA MIKONO SAFI, MKOA WA KAGERA

KIJARIDA CHA MAELEZO KWA WAZAZI

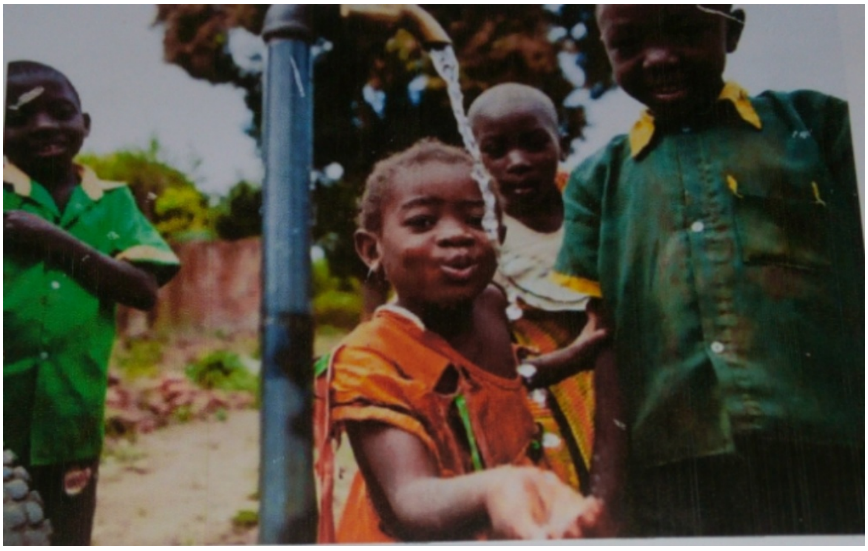

Kwa nini tuko hapa?

- Tunashirikiana na idara ya elimu katika Halmashauri ya Manispaa ya Bukoba mjini ili kuboresha tabia ya kunawa mikono kwa kutumia sabuni na maji kwa watoto wa shule za msingi katika jamii yako. Hii itasaidia kuwakinga watoto wenu na maambukizi ya minyoo ya tumboni na kuboresha afya zao.
- Tukishirikiana na Halmashauri ya Manispaa ya Bukoba mjini, tumewapima minyoo ya tumboni baadhi ya watoto wa shule za msingi. Utayakuta majibu ya vipimo vya mwanao kwenye bahasha nyingine.

Kuna athari gani kama mtoto wako ana minyoo?

- Kama mwanao ana minyoo, minyoo inaweza kunyonya damu yake mwilini na kupelekea mtoto wako kuwa na kiwango kidogo cha damu tofauti na kiwango kinachostahili.
- Minyoo nayo huwa inatumia chakula kilichopo tumboni baada ya mtoto wako kula. Hii inaweza kusababisha mtoto wako kupata utapiamlo maana chakula kingine kinakuwa kimeliwa na minyoo.

- Minyoo humfanya mtoto kuchoka sana, na hali hii inamfanya mtoto asiwe anafanya vizuri shuleni na hata nyumbani.
- Kama mtoto akiwa na minyoo mingi sana anaweza kuhitaji kufanyiwa upasuaji wa tumboni.

Ni kwa namna gani mtoto anaweza kupata minyoo?

- Watoto wanaweza kuambukizwa minyoo kwa njia mbalimbali. Kwa mfano watoto wanaweza kupata minyoo iwapo hawatonawa mikono yao kwa maji safi na sabuni baada ya kutoka chooni au kama watakula chakula bila kunawa mikono kwanza.
- Baadhi ya minyoo huzaliana kwenye udongo na vimelea vya minyoo huingia mwilini kwa kutoboa ngozi ya mwili. Hivyo kama mtoto wako atatembea bila viatu anaweza kupata maambukizi ya minyoo kwa urahisi zaidi.
- Mayai ya minyoo huweza kuishi kwenye uchafu, kwenye mikono ya mtu iwapo ataenda chooni kisha asinawe mikono yake kwa maji na sabuni, matunda au mbogamboga zisizosafishwa vizuri. Kama mtoto wako atachezea uchafu, atakula matunda ambayo hayajaoshwa au atashika mikono michafu ya mtu mwingine na kuweka vidole vyake mdomoni mwake atapata minyoo.
- Kwa vile huwezi kuona mayai ya minyoo kwa macho haimaanishi kwamba hayapo. Mayai ya minyoo ni madogo sana huwezi kuyaona mpaka utumie kifaa maalumu kama darubini.

Majibu ya mwanangu ya vipimo vya minyoo yanamaanisha nini?

- Karatasi iliyoambatanishwa inaonyesha aina na kiasi cha maambukizi ya minyoo kwa kila kiwango cha uzito (katika gramu)wa kinyesi tuliyochopima. Maelezo na namba zilizotolewa zinaweza kuwa ngumu kueleweka,Ili kurahisisha tumetumia mfumo wa rangi.
- Utaona kwamba kuna aina nne za minyoo ambazo tutapima na kwa kila aina ya mnyoo majibu yataonyeshwa kwa kutumia rangi ya kijani au nyekundu.

- Kwa kila aina ya mnyoo utakaopimwa, kama mtoto wako hana maambukizi rangi itakayoonekana ni ya kijani. Iwapo mtoto wako ana maambukizi rangi itakayoonekana ni nyekundu. Kama mtoto wako ana maambukizi ndani ya sanduku la rangi nyekundu chini ya jina la mnyoo kutakuwa na namba ambayo inaonyesha idadi ya mayai ya minyoo kwa kila gramu ya choo kilichopimwa. Namba hii ikiwa kubwa maana yake mtoto wako ana mayai mengi sana kwenye choo, ikionyesha kuwa ana maambukizi makubwa zaidi ya minyoo mwilini. Kama mtoto wako hana minyoo, hakuta kuwa na namba yeyote ndani ya kisanduku cha rangi ya kijani.

Hakuna maambukizi	Ana maambukizi

Kwa mfano: Majibu yafuatayo hapa chini yanaonyesha hakuna maambukizi kwenye safu ya kwanza na ya tatu (rangi ya kijani), na kuna maambukizi kwenye safu ya pili na ya nne (rangi nyekundu). Kiwango cha maambukizi ni kikubwa zaidi kwenye safu ya nne kuliko safu ya pili.

S. mansoni	Hookworm	Ascaris	Trichuris
	890		6700

Ni nini kitafanyika ikiwa mtoto wangu atapatikana na minyoo?

- Kama sehemu ya mpango huu wa utafiti, tutawapatia dawa watoto wote ambao watapatikana na minyoo. Hii haitakugharimu kitu, watoto hawa watapewa dawa bure.
- Hata hivyo, kuna hatua unazoweza kuchukua nyumbani kwako ili kumkinga mtoto wako na maambukizi ya minyoo kwa siku zijazo.

Nifanye nini nyumbani kwangu ili kuwa kinga watoto wangu na maambukizi ya minyoo?

- Hakikisha kuwa wanaweza kunawa mikono pale nyumbani.
    - Weka ndoo ya maji, sabuni na jagi la kuchotea maji karibu na chooni kwa ajili ya kunawa mikono.

    - Hakikisha vifaa hivi vyote viko karibu na choo sehemu ambayo watoto/mtoto anaweza kuviona akiwa anatoka chooni.
- Wahimize watoto wako kunawa mikono.
    - Fanya mazungumzo na watoto/mtoto kuhusu kunawa mikono. Waambie wakuonyeshe namna wanavyo nawa mikono.
    - Kila wakati unapomuona mtoto anaenda chooni mkumbushe kuhusu kunawa mikono.
    - Kila wakati mtoto anapotaka kula, hata kama ni kitafunio cha chai au soda, hakikisha ananawa mikono yake.
    - Fanya majadiliano na watoto pamoja na watu walioko kwenye kaya yako kuhusu umuhimu wa kunawa mikono.
- Kila wakati osha matunda kwa maji safi kabla ya kula.
- Tumia maji yanayotoka kwenye vyanzo salama. Chemsha au weka dawa maji pale nyumbani ili kuhakikisha ni salama. Hata maji unayoyaona ni masafi yanaweza kuwa na mayai au vimelea vya minyoo
- Kuwa mfano wa kuigwa**!** Watoto hujifunza kwa kuangalia watu wengine wanachofanya. Ukiwa mfano wa kuigwa kwa watoto kutawahimiza watoto kunawa mikono.

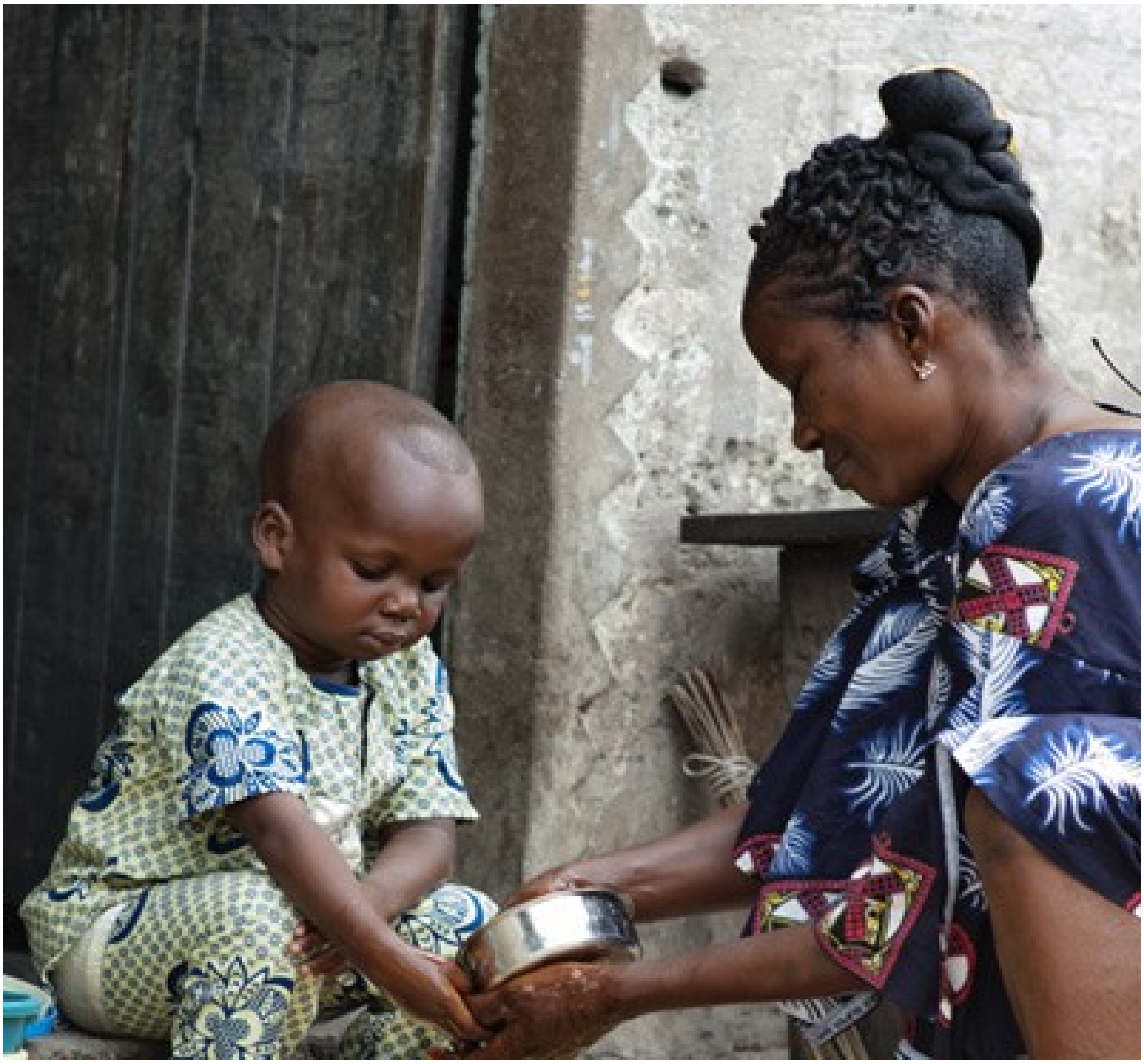

Nawa mikono kwa usahihi kwa kufuata hatua zinazoonyeshwa katika picha hii hapa chini

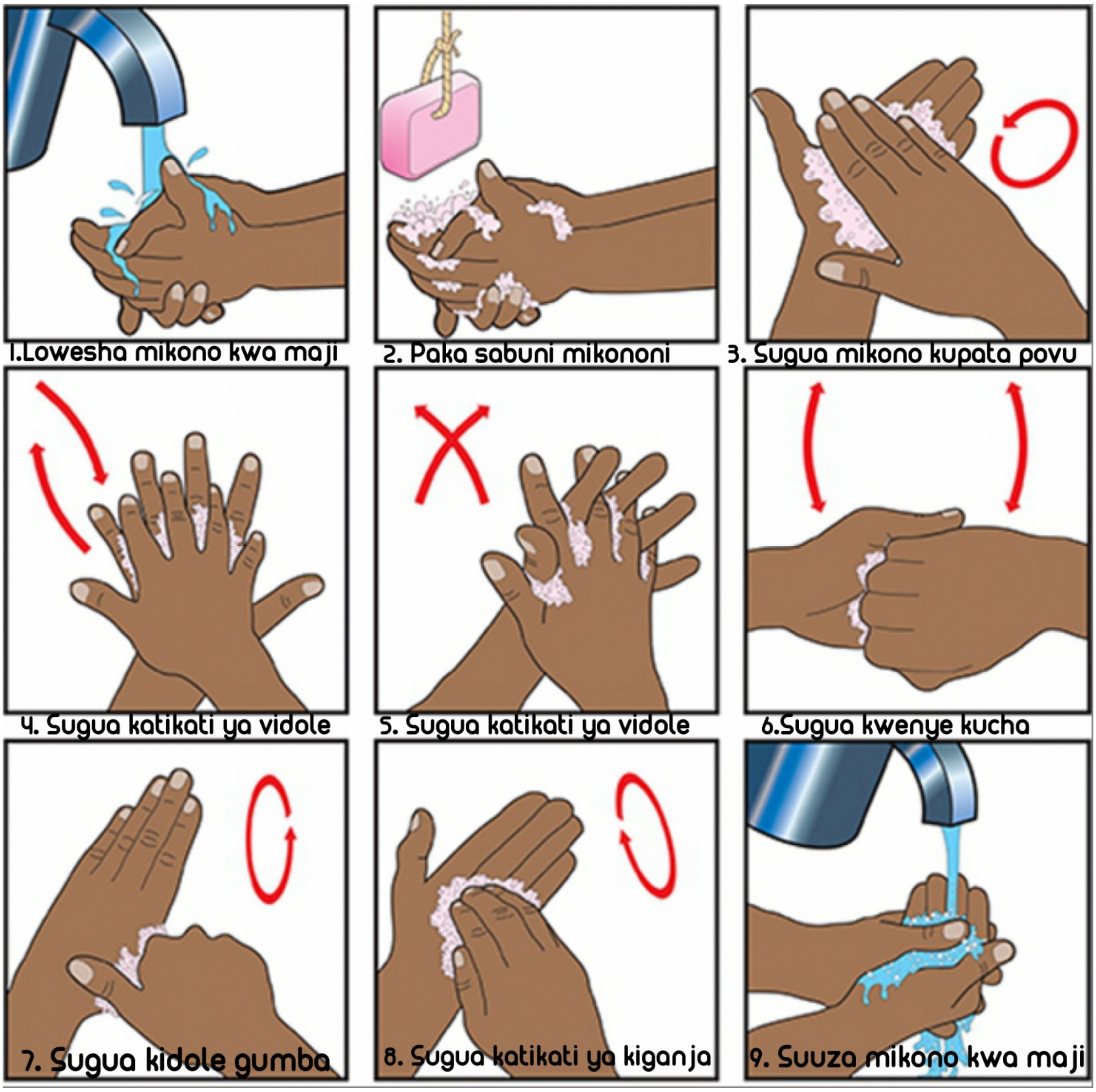

Supplement: S2 Appendix — (PDF) [file pone.0242240.s002.pdf]
