# Supplementary material for: Hand hygiene intervention to optimize helminth infection control: Design and baseline results of Mikono Safi–An ongoing school-based cluster-randomised controlled trial in NW Tanzania
Source: PLoS One. 2020 Dec 9;15(12):e0242240. doi: 10.1371/journal.pone.0242240 (PMC7725373; doi:10.1371/journal.pone.0242240)
Supplement: S3 Appendix — (PDF) [file pone.0242240.s003.pdf]

Mikono Safi Curriculum

Target objectives:

- Children will learn the importance of handwashing with soap and how to wash properly.
- Emotional and motivational drivers will be used to drive behavior change in the children.

Learning materials used:

- Board game
- Side by side poster
- Koku and Muta story
- Steps to handwashing poster
- Handwashing song/Paulo song
- Ball game about the spread of germs

Activities:

1. **Kickoff Event**
   There will be **one** Kickoff Event that incorporates multiple activities and helps give the children a basis of knowledge on proper handwashing behaviors, introduce new intervention hardware and materials, and targeted behavior change activities designed to leverage behavior change

2. **Booster Sessions**
   There will be **two** booster sessions spaced throughout the year that incorporate the materials and lessons from field day, while also building on key points such as emotional drivers and social norms.

KICKOFF EVENT 3

Kickoff Activity 1: Side by side poster 4
Kickoff Activity 2: Koku and Muta story 5
Kickoff Activity 3: Handwashing steps poster 6
Kickoff Activity 4: Germ spreading game 7
Kickoff Activity 5: Commitment ceremony 8

BOOSTER SESSION 1 9

Booster Session 1, Activity 1: Koku and Muta story 10
Booster Session 1, Activity 2: The "Paulo una mikono michafu" song 11
Booster Session 1, Activity 3: Handwashing Board Game 12
Booster Session 1, Activity 4: Germ spreading game 13

BOOSTER SESSION 2 14

Booster Session 2, Activity 1: Handwashing steps poster 15
Booster Session 2, Activity 2: Side by side poster 16
Booster Session 2, Activity 3: Food contamination game 17

Kickoff Event

Goals of this activity:

- To familiarize children with the learning materials
- To teach children proper handwashing steps
- To touch on the children's emotional drivers to drive behavior change
- To introduce children to the environmental modifications and nudges that have been incorporated into the school grounds
- To have a practical demonstration of hand washing with soap

Key points that should be mentioned during the Kickoff Event:

- Emotional drivers such as fear, disgust, nurture, etc.
- Important times for handwashing, i.e. after latrine use and before eating
- Hands should not only be washed when they appear dirty, they should be washed before and after key events
- Effective hand washing should always involve use of soap

How to conduct the Kickoff Event:

- Create a schedule for which classes will be taught on which days, and designate one teacher per class (ex: Monday standard 3, Tuesday standard 4, etc).
- Set aside a 40-minute class session each day to conduct the field day
- Familiarize yourself with the learning materials before the field day so you can explain them to the students and answer any questions that come up
- The field day will include a session of in-class teaching, as well as practical examples outside
- Schedule two teacher's aides to come help with the day's activities

Activities include:

1. **Side-by-side poster discussion**
2. **Koku and Muta Story (with flip chart)**
3. **Handwashing steps poster and demonstration of new handwashing stations**
4. **Germ Spreading Game**
5. **Commitment Ceremony**

Kickoff Activity 1: Side by side poster

Description of the material: on the left side, the boy exits the latrine and gets a snack without washing her hands, on the right side the girl exits the latrine, washes her hands with soap and water, and then eats a snack

Activities

- Hang the poster up in the front of the classroom
- Ask the children to describe what they see in the poster and how they feel about what they see
- Point out to the children that by not washing her hands and then eating a snack, the girl ingested the poop or pee that was on her hands from going to the latrine
- Talk about how on the side where the boy did not wash her hands, his stomach is infested with worms, and on the side where the girl did wash her hands, her stomach is worm free
- Ask the children what the boy should have done differently so he would not get worms

Kickoff Activity 2: Koku and Muta story

Description of the material: this is a seven-page flipchart with images of a boy and a girl having a discussion on it. The boy is carrying a shield and the girl is asking him why he has it

Activities:

- Use the easel to hang the Koku and Muta story at the front of the classroom
- Call two students up to the front, one boy and one girl. The boy will read the part of Muta, the girl will read the part of Koku. Tell the boy he can use a silly voice or gestures when reading the part of Muta
- After each page, discuss with the students what happened and what it means. For example, on the page where Koku says it is disgusting that Muta does not use soap, focus on the disgust aspect and why it is bad that Muta does not wash his hands after using the latrine. Emphasize that Muta will be eating dirt or feces if he eats without washing his hands. Mention that the reason Muta was holding the shield was that he was afraid of what diseases ("invaders") might enter his body
- When the story is over, the two students can sit back down. Have a class-wide discussion about how Muta changed his behavior and why it helped his health

Kickoff Activity 3: Handwashing steps poster

Description of the material: This is a poster with five squares that show the steps to handwashing.

Activities:

- Hang the handwashing steps poster at the front of the classroom
- Go through each square of the poster: in the first square, the children are going from the latrine to the hand washing station, in the second square, the child is applying soap on his hands, in the third square, the child rubs his palms together, in the fourth square, the child rubs in between his fingers, and in the last square the child rinses his hands with clean water from the hand washing stand. Be sure to tell the children that when they are rubbing their hands together they must count to ten
- Once you have gone through all of the steps, teach this song to the children and sing it as a class:

Sasa ni wakati mzuri kwenda kujisaidia
Baada ya kutoka chooni, tunawe mikono yetu
Tunawe mikono kwa sabuni
Tunawe mikono kwa maji
Mikono yetu iwe safi tuepuke magonjwa

Kabla ya kula chakula tunawe mikono yetu
Kabla ya kula matunda tuyaoshe kwa maji
Mtoto mzuri hukumbuka kunawa mikono yake baada ya kutoka chooni pia kabla ya kula.

- After you have taught the children the song, go outside to the handwashing stations
- Show the children how to wash their hands, being sure to use soap, rub hands together until suds form and then count to ten, and then rinse hands with clean water
- After you have demonstrated to the children how to wash their hands, call a few children up to wash their hands in front of other students. Make sure they do it properly and discuss with the class how they did it

Kickoff Activity 4: Germ spreading game

Description of the materials: this game requires a soccer ball and chalk dust

Activities

- Stay outside after conducting the practical handwashing lesson
- Divide the class into groups of ten children
- Give each group a soccer ball and tell them to hold onto it
- Have the teacher's aides go to each group and turn away from the group and spread some chalk dust on the ball so the kids do not see it
- Tell the children to play catch, throwing the ball around the group of ten kids
- After a few minutes, tell the children to look at their hands. They will see that chalk dust has spread throughout the group of children.
- When children see chalk on their hands, talk about how germs spread and how if their classmate went to the bathroom before playing and did not wash his/her hands with soap, that could have been poop moving around (disgust).

Kickoff Activity 5: Commitment ceremony

Description of the activity: this activity serves to get children to say in front of their class that they will stay committed to the program and will continue to wash their hands

Activities:

- Go back into the classroom with your entire class
- Ask children who wants to commit to washing their hands with soap and water after using the latrine and before eating
- It might take some nudging to get them to come up, so you might need to choose a few
- When children say they want to commit, bring them to the front of the classroom and have them stand facing their classmates
- Have the children repeat after you: ** work with teachers on a script for the pledge

I will wash my hands after using the latrine
I will wash my hands before eating
I will use soap and water every time
Because of this, I will be healthy and happy

- Once the children have finished repeating the pledge, they may sit down again
- Ask if any other children want to come up, and repeat the process

Booster Session 1

Goals of this activity:

- To remind children about reasons for handwashing
- To re-familiarize children with the educational materials

Key points that must be mentioned:

- Emotional drivers such as fear, disgust, nurture, etc.
- Important times for handwashing, i.e. after latrine use and before eating

How to conduct a booster session:

- Create a schedule for which classes will be taught on which days, and designate two teachers per class
- Set aside a 40-minute class session to conduct the field day
- Review the learning materials before the lesson so you can explain them to the students and answer any questions that come up

Activities included in the booster session:

1. **Booster 1: Koku and Muta Story**
2. **The “Paulo una mikono michafu” song**
3. **Handwashing Board Game**
4. **Germ Spreading Game**

Booster Session 1, Activity 1: Koku and Muta story

Description of the material: this is a seven-page flipchart with images of a boy and a girl having a discussion on it. The boy is carrying a shield and the girl is asking him why he has it

Activities:

- Use the easel to hang the Koku and Muta story at the front of the classroom
- Call two students up to the front, one boy and one girl. The boy will read the part of Muta, the girl will read the part of Koku. Tell the boy he can use a silly voice or gestures when reading the part of Muta
- After each page, discuss with the students what happened and what it means. For example, on the page where Koku says it is disgusting that Muta does not use soap, focus on the disgust aspect and why it is bad that Muta does not wash his hands after using the latrine. Emphasize that Muta will be eating dirt or feces if he eats without washing his hands. Mention that the reason Muta was holding the shield was that he was afraid of what diseases ("invaders") might enter his body
- When the story is over, the two students can sit back down. Have a class-wide discussion about how Muta changed his behavior and why it helped his health

Booster Session 1, Activity 2: The "Paulo una mikono michafu" song

Description of this activity: sing a song about hand washing with the class

Activities:

- Talk through the lyrics with the children before singing it to make sure everyone knows the words

  Paulo usije kucheza na sisi una mikono michafu,
  Tutachafuka hata kunuka, una mikono michaafu
  Paulo usije kucheza na sisi una mikono michaafu
  Hutaki kunawa sabuni ndiyo dawa, una mikono michaafu
  Paulo usije kula na sisi una mikono michaafu,
  Hutaki kunawa sabuni ndiyo dawa, unamikono michaafu

- After you sing the song, talk to the class about why no one wanted to play with Paulo and how a behavior change could have helped him make friends

Booster Session 1, Activity 3: Handwashing Board Game

Description of the materials needed for this game: board game, clean hand tokens, playing pieces

Activities:

- Hold a board game up in front of the class and explain how to play
- Each child gets five “clean hand” tokens to start with
- Each round can be played by about 5 students
- Each child places a playing piece on the “anziahapa” square
- One child spins the spinner, and either moves forward the number of spaces on the spinner or picks up a card from the pile
- If the child picks up a card, they should read what it says and do what it says at the bottom
- Any time a child lands on a space, they should either add or take away the number of clean hand tokens it says
- Once a child’s turn is over, the next child goes
- The game continues until all children have reached the “mwisho” square
- Once everyone has finished, each child should count how many clean hand tokens they have. The child with the most wins
- Once all of the groups have finished playing, discuss with the class which squares were the best and worst to land on and why

Booster Session 1, Activity 4: Germ spreading game

Description of the materials: this game requires a soccer ball and chalk dust

Activities:

- Stay outside after conducting the practical hand washing lesson
- Divide the class into groups of ten children
- Give each group a soccer ball and tell them to hold onto it
- Have the teacher's aides go to each group and turn away from the group and spread some chalk dust on the ball so the kids do not see it
- Tell the children to play catch, throwing the ball around the group of ten kids
- After a few minutes, tell the children to look at their hands. They will see that chalk dust has spread throughout the group of children.
- When children see chalk on their hands, talk about how germs spread and how if their classmate went to the bathroom before playing and did not wash his/her hands with soap, that could have been poop moving around (disgust).

Booster Session 2

Goals of this activity:

- To remind children about proper handwashing technique
- To re-familiarize children with the educational materials

Key points that must be mentioned:

- Emotional drivers such as fear, disgust, nurture, etc.
- Important times for handwashing, i.e. after latrine use and before eating
- Proper steps for handwashing

How to conduct a booster session:

- Create a schedule for which classes will be taught on which days, and designate two teachers per class
- Set aside a 40-minute class session to conduct the field day
- Review the learning materials before the booster so you can explain them to the students and answer any questions that come up

Activities included in the booster session:

1. **Handwashing steps poster**
2. **Side-by-side poster**
3. **Food contamination game**

Booster Session 2, Activity 1: Handwashing steps poster

Description of the material: This is a poster with five squares that show the steps to hand washing.

Activities:

- Hang the handwashing steps poster at the front of the classroom
- Go through each square of the poster: in the first square, the children are going from the latrine to the hand washing station, in the second square, the child is applying soap on his hands, in the third square, the child rubs his palms together, in the fourth square, the child rubs in between his fingers, and in the last square the child rinses his hands with clean water from the hand washing stand. Be sure to tell the children that when they are rubbing their hands together they must count to ten
- Once you have gone through all of the steps, teach this song to the children and sing it as a class:

Sasa ni wakati mzuri kwenda kujisaidia
Baada ya kutoka chooni, tunawe mikono yetu
Tunawe mikono kwa sabuni
Tunawe mikono kwa maji
Mikono yetu iwe safi tuepuke magonjwa

Kabla ya kula chakula tunawe mikono yetu
Kabla ya kula matunda tuyaoshe kwa maji

Mtoto mzuri ukumbuka kunawa mikono yake baada ya kutoka chooni pia kabla ya kula

- After you have taught the children the song, go outside to the hand washing stations
- Show the children how to wash their hands, being sure to use soap, rub hands together until suds form and then count to ten, and then rinse hands with clean water
- After you have demonstrated to the children how to wash their hands, call a few children up to wash their hands in front of other students. Make sure they do it properly and discuss with the class how they did it

Booster Session 2, Activity 2: Side by side poster

Description of the material: on the left side, the boy exits the latrine and gets a snack without washing her hands, on the right side the girl exits the latrine, washes her hands with soap and water, and then eats a snack

Activities:

- Hang the poster up in the front of the classroom
- Ask the children to describe what they see in the poster and how they feel about what they see
- Point out to the children that by not washing his hands and then eating a snack, the boy ingested the poop or pee that was on his hands from going to the bathroom
- Talk about how on the side where the boy did not wash his hands, his stomach is infested with worms, and on the side where the girl did wash her hands, her stomach is worm free
- Ask the children what the boy should have done differently so she would not get worms

Booster Session 2, Activity 3: Food contamination game

Description of the materials: for this game, you need a snack, a stick, and some mud

Activities:

- Take the snack that has been given to you by the research team and gather your class in a group outside
- Select two students to come to the front of the group with you
- Ask the students to take a bite out of the snack
- After the children have taken a bite out of the snack, take a stick and rub it in mud
- Rub a small amount of the mud on the snack
- Ask the children if they can see the mud. They should not be able to because it is a very small amount
- Ask the children if they want to take another bite out of the snack.
- Once the children say no, ask them why they did not want to eat anymore of the snack
- After they give their answers, talk to the class about how even though they could not necessarily see the mud, it was on the food and therefore the food should not be eaten
- Explain that when kids go to the bathroom without washing their hands, the same thing can happen to their food. The mud (poop) can get on their hands in the latrine, and then when they grab a snack it will move to the food even if they cannot see it
