# Supplementary material for: Hand hygiene intervention to optimize helminth infection control: Design and baseline results of Mikono Safi–An ongoing school-based cluster-randomised controlled trial in NW Tanzania
Source: PLoS One. 2020 Dec 9;15(12):e0242240. doi: 10.1371/journal.pone.0242240 (PMC7725373; doi:10.1371/journal.pone.0242240)
Supplement: S4 Appendix — (PDF) [file pone.0242240.s004.pdf]

Mtaala wa mikono safi

Malengo yanayokusudiwa:

- Watoto watajifunza umuhimu wa kunawa mikono kwa kutumia sabuni na namna ya kunawa mikono kwa usahihi
- Vichocheo vya kihisia na motisha vitatumika ili kuweza kuchochea mabadiliko ya tabia kwa watoto

Zana zitakazotumika kujifunzia:

- Mchezo wa kadi unaohusu kunawa mikono
- Bango lenye sehemu mbili linaloonyesha minyoo inavyo sambazwa
- Chati yenye hadithi ya Koku na Muta
- Zana yenye hatua za kunawa mikono
- Wimbo wa Paulo una mikono michafu
- Mchezo wa mpira wa namna vimelea vya maradhi husambaa

Mambo ya kufanya

1. **Kipindi cha kwanza**
   Kutakuwa na kipindi **kimoja** ambacho kitajumuisha shughuli mbalimbali ili kusaidia kuwapatia watoto msingi bora wa maarifa kuhusu tabia bora ya kunawa mikono, kipindi hiki pia kitahusisha kuweka vifaa vipya vya kunawa mikono, pamoja na kufanya shughuli mbalimbali zinazolenga kuchochea mabadiliko ya tabia.

2. **Kipindi cha marudio**
   Kutakuwa na vipindi **viwili** vya marudio vitakavyofanyika kwa mwaka mzima. Vipindi hivyo vitahusisha matumizi ya zana na masomo yaliyotangulia kufundishwa katika kipindi cha kwanza, pia vitaendelea kugusia mambo muhimu kama vichocheo vya kihisia na mambo yanayokubalika na jamii kiujumla kuhusu usafi.

MTAALA WA MIKONO SAFI 1

KIPINDI CHA KWANZA 3

KIPINDI CHA KWANZA, ZOEZI LA 1: BANGO LENYE SEHEMU MBILI LINALOONYESHA NAMNA MINYOO INAVYOENEZWA 4
KIPINDI CHA KWANZA, ZOEZI LA 2: HADITHI YA KOKU NA MUTA 4
KIPINDI CHA KWANZA, ZOEZI LA 3: BANGO LINALOONYESHA HATUA ZA KUNAWA MIKONO 5
KIPINDI CHA KWANZA, ZOEZI LA 4: MCHEZO WA KUONYESHA NAMNA VIMELEA VYA MARADHI HUENEZWA 6
KIPINDI CHA KWANZA, ZOEZI LA 5: KIAPO CHA AHADI YA KUDUMISHA MAZOEA BORA YA KUNAWA MIKONO 6

KIPINDI CHA MARUDIO CHA 1 8

KIPINDI CHA KWANZA CHA MARUDIO, ZOEZI LA 1: HADITHI YA KOKU NA MUTA 8
KIPINDI CHA KWANZA CHA MARUDIO, ZOEZI LA 2: WIMBO WA "PAULO UNA MIKONO MICHAFU" 9
KIPINDI CHA KWANZA CHA MARUDIO, ZOEZI LA 3: MCHEZO WA KADI KUHUSU KUNAWA MIKONO 9
KIPINDI CHA KWANZA CHA MARUDIO, ZOEZI LA 4: MCHEZO WA NAMNA VIMELEA VYA MARADHI HUSAMBAA 10

KIPINDI CHA MARUDIO CHA 2 11

KIPINDI CHA PILI CHA MARUDIO, ZOEZI LA 1: BANGO LINALOONYESHA HATUA ZA KUNAWA MIKONO 12
KIPINDI CHA MARUDIO CHA 2, ZOEZI LA 2: BANGO LENYE SEHEMU MBILI KUHUSU NAMNA MINYOO INAVYOENEZWA 13
KIPINDI CHA MARUDIO CHA 2, ZOEZI LA 3: MCHEZO WA UCHAFUZI WA CHAKULA 13

Kipindi cha kwanza

Malengo ya kipindi:

- kuzoeza watoto zana za kujifunzia
- Kuwafundisha watoto hatua sahihi za kunawa mikono
- Kugusa vichocheo vya kihisia vya watoto ili kuchochea mabadiliko ya tabia
- Kuwaoyesha na kuwatambulisha watoto maboresho ya kimazingira na "*michoro-alama"* ambayo imewekwa kwenye maeneo ya shule

Mambo muhimu ambayo ni lazima yagusiwe kwenye kipindi cha kwanza:

- Vichocheo vya kihisia kama hofu, inayochukiza na makuzi, n.k.
- Nyakati muhimu za kunawa mikono ambazo ni baada ya kutoka chooni na kabla ya kula
- Sio lazima mikono ionekane michafu ndio unawe, ila unatakiwa kunawa mikono kabla na baada ya matukio muhimu
- Ufanisi katika kunawa mikono ni lazima kutumia sabuni

Namna ya kuandaa kipindi cha kwanza:

- Andaa ratiba kuonyesha ni madarasa yapi yatafundishwa katika siku gani, pia panga walimu wawili kwa darasa (mfano, Jumatatu darasa la 3, Jumanne darasa la 4, n.k).
- Tenga dakika 40 kwa siku kufundisha kipindi hiki cha kwanza kwa kila darasa
- Pitia zana utakazotumia kufundishia kabla ya kipindi ili uweze kuzielewa na kuweza kuwaelezea wanafunzi na kujibu maswali yote yatakayoulizwa
- Kipindi hiki kitajumuisha mafunzo ya darasani pamoja na mafunzo ya vitendo nje ya darasa
- Andaa walimu wawili watakaosaidia shuguli za siku hii

Shughuli za kufanya:

1. **Bango lenye sehemu mbili linaloonyesha namna minyoo inavyoenezwa**
2. **Hadithi ya Koku na Muta**
3. **Bango linaloonyesha hatua za kunawa mikono na kuelekeza kwa vitendo matumizi ya sehemu mpya za kunawa mikono**
4. **Mchezo wa namna vimelea vya maradhi vinavyoenea**
5. **kiapo cha ahadi ya kudumisha tabia bora ya kunawa mikono**

Kipindi cha kwanza, zoezi la 1: Bango lenye sehemu mbili linaloonyesha namna minyoo inavyoenezwa

Maelezo ya zana: Unapotazama bango upande wa kushoto unaonyesha mvulana anatoka chooni na kula kitafunwa bila kunawa mikono, upande wa kulia unaonyesha msichana anatoka chooni na kunawa mikono yake kwa maji na sabuni kisha anakula kitafunwa

Shughuli za kufanya

- Tundika bango moja mbele ya darasa na mengine mawili, kila moja kwenye kuta za pembeni. Waulize watoto waeleze ni nini wanakiona katika bango hilo na wanahisije kutokanana wanachokiona.
- Waelezee watoto kuwa kutokana na kula kitafunwa bila kunawa mikono huyu mvulana amekula kinyesi au mkojo ambao ulikuwa kwenye mikono yake alipotoka chooni
- Zungumzia upande ambao mvulana hakunawa mikono yake namna ameshambuliwa na minyoo tumboni mwake, na kwa upande wa msichana ambaye alinawa mikono yake hana minyoo tumboni mwake
- Waulize watoto ni kitu gani cha tofauti huyu mvulana angefanya ili asipate minyoo

Kipindi cha kwanza, zoezi la 2: Hadithi ya Koku na Muta

Maelekezo ya zana: Bango hili lina kurasa saba zenye picha ya mvulana na msichana wakifanya mazungumzo. Mvulana amebeba ngao na msichana anamuuliza kwanini ameibeba ngao.

Shughuli za kufanya:

- Tumia stendi ya kutundikia kuonyesha bango lenye hadithi ya Koku na Muta mbele ya darasa
- Chagua wanafunzi wawili waje mbele ya darasa, mmoja mvulana na mmoja msichana. Mvulana atasoma sehemu ya Muta na msichana atasoma sehemu ya Koku. Mwambie mvulana anaweza kutumia sauti ya kuchekesha wakati anasoma sehemu ya Muta.
- Katika kila kurasa, jadili na wanafunzi kilichotokea katika ukurasa huo na inamaanisha nini. Mfano, kwenye kurasa ambayo Koku anasema inachukiza Muta hatumii sabuni, weka ***mkazo kwenye kipengele cha kuchukiza*** na ni kwanini ni jambo baya Muta hanawi mikono yake kwa sabuni na maji baada ya kutoka chooni. Sisitiza kuwa Muta atakuwa anakula uchafu au kinyesi iwapo hatanawa mikono kwa

maji na sabuni. Waeleze sababu ya Muta kushika ngao ni kuwa anaogopa kuwa kuna magonjwa ("*wavamizi*") yanaweza kuingia kwenye mwili wake.

- Baada ya hadithi kuisha, wale wanafunzi wawili wanaweza kurudi kukaa. Fanya majadiliano na darasa zima ni namna gani Muta amebadilisha tabia na ni kwanini imemsaidia kiafya.

Kipindi cha kwanza, zoezi la 3: Bango linaloonyesha hatua za kunawa mikono

Maelezo ya zana: Hili ni bango lenye visanduku vitano vinavyoonyesha hatua za kunawa mikono.

Shughuli za kufanya:

- Tundika bango moja linaloonyesha hatua za kunawa mikono mbele ya darasa na mengine mawili, kila moja kwenye katika kuta mbili za pembeni angalau karibu na katikati ya darasa.
- Elezea kila kisanduku cha bango: kwenye kisanduku cha kwanza mtoto anatoka chooni na kwenda sehemu ya kunawia mikono, kwenye kisanduku cha pili, mtoto analowesha mikono kwa maji na kupaka sabuni kwenye mikono yake, kisanduku cha tatu anasugua mikono yake kwa pamoja, kisanduku cha nne anasugua katikati ya vidole na kwenye kisanduku cha mwisho mtoto anasuuza mikono yake kwa maji safi kutoka kwenye tanki la kunawia mikono. Hakikisha unawaambia watoto wakati wanasugua mikono yao kwa pamoja wanatakiwa kuhesabu hadi kumi.
- Baada ya kumaliza kuelezea hatua zote za kunawa mikono, wafundishe watoto wimbo huu na muuimbe darasa zima:

Sasa ni wakati mzuri kwenda kujisaidia

Baada ya kutoka chooni, tunawe mikono yetu

Tunawe mikono kwa sabuni

Tunawe mikono kwa maji

Mikono yetu iwe safi tuepuke magonjwa

Kabla ya kula chakula tunawe mikono yetu

Kabla ya kula matunda tuyaoshe kwa maji

Mtoto mzuri ukumbuka kunawa mikono yake

baada ya kutoka chooni pia kabla ya kula

- Baada ya kuwafundisha watoto wimbo nendeni nje kwenye sehemu za kunawia mikono
- Waonyeshe watoto namna ya kunawa mikono yao, hakikisha unatumia sabuni, sugua mikono kwa pamoja mpaka uone povu kisha hesabu hadi kumi, kisha suuza mikono yako kwa maji safi

- Baada ya kuwaonyesha watoto namna ya kunawa mikono, waite watoto wachache ili wanawe mikono mbele ya wanafunzi wengine. Hakikisha wananawa kwa usahihi na kisha fanya majadiliano kuelezea namna walivyofanya.

Kipindi cha kwanza, zoezi la 4: **Mchezo wa kuonyesha namna vimelea vya maradhi huenezwa**

Maelezo ya vifaa vinavyohitajika katika mchezo huu: Mchezo huu unahitaji mpira na vumbi la chaki

Shughuli za kufanya:

- Baada ya kumaliza kuwaelekeza wanafunzi namna ya kunawa mikono bakini nje ya darasa kwa muda mfupi
- Gawanya wanafunzi kwenye makundi kila kundi liwe na wanafunzi kumi
- Wape kila kikundi mpira mmoja na uwaambie waushike
- Mwombe mwalimu mmoja msaada wa kwenda kwenye kila kundi kisha achukue mpira na kugeukia sehemu ambayo wanafunzi hawatamuona kisha apake vumbi la chaki kwenye mpira
- Waambie wanafunzi warushiane mpira, hakikisha kila mwanafunzi kwenye kikundi ameshika mpira
- Baada ya dakika chache, waambie wanafunzi waangalie mikono yao. Wataona kuwa vumbi la chaki limeenea kwenye kundi zima la watoto.
- Baada ya watoto kuona chaki kwenye mikono yao, zungumzia namna vimelea vinaenea kwa kutumia mfano huo wa chaki, na pia ikiwa mwenzao angeenda chooni na akatoka bila ya kunawa mikono kwa sabuni alama hizo za chaki zingekuwa ni kinyesi ambacho kingeenea kwao wote ***(inachukiza).***

Kipindi cha kwanza, zoezi la 5: Kiapo cha ahadi ya kudumisha mazoea bora ya kunawa mikono

Maelezo ya zoezi: Zoezi hili linakusudia watoto waseme mbele ya darasa kwamba wanaahidi na wataendelea kunawa mikono yao.

Shughuli za kufanya*:*

- Rudi darasani pamoja na wanafunzi wote
- Waulize watoto nani anataka kuahidi kunawa mikono yake kwa maji na sabuni baada ya kutoka chooni na kabla ya kula
- Inawezekana ikachukua muda kuwashawishi wanafunzi kujitokeza, hivyo unaweza kuhitaji kuchagua wanafunzi wachache
- Watoto wakisema wanataka kuahidi, waambie waje mbele ya darasa kisha wasimame na kuwageukia wanafunzi wenzao

- Waambie watoto warudie unachosema: (Walimu wanaweza kutoa mapendekezo ya maneno ya kiapo)

Nitanawa mikono yangu baada ya kutoka chooni

Nitanawa mikono yangu kabla ya kula

Nitatumia sabuni na maji wakati wote

Kwasababu hii nitakuwa mwenye afya na furaha

- Baada ya watoto kumaliza kusema kiapo, wanaweza kwenda kukaa
- Uliza kama kuna watoto wengine wanataka kuja mbele, kisha rudia zoezi

Kipindi cha marudio cha 1

Malengo ya kipindi hiki:

- Kuwakumbusha watoto sababu za kunawa mikono
- Kuwazoeza kwa mara nyingine watoto mambo yaliyomo kwenye zana za kujifunzia

Mambo muhimu ya kuongelea:
- Vichocheo vya kihisia kama hofu, yenye kuchukiza, makuzi, n.k.
- Nyakati muhimu za kunawa mikono, mfano baada ya kutoka chooni na kabla ya kula

Namna ya kuandaa kipindi:

- Andaa ratiba kuonyesha ni madarasa yapi yatafundishwa katika siku gani, pia panga walimu wawili kwa darasa
- Tenga dakika 40 kwa siku kufundisha kipindi hiki cha kwanza kwa kila darasa
- Pitia zana utakazotumia kufundishia kabla ya kipindi ili uweze kuzielewa na kuweza kuwaelezea wanafunzi na kujibu maswali yote yatakayoulizwa

Shughuli za kufanya
1. **Hadithi ya Koku na Muta**
2. **Wimbo wa "*Paulo una mikono michafu*"**
3. **Mchezo wa kadi unaohusu kunawa mikono**
4. **Mchezo wa namna vimelea vya maradhi husambaa**

Kipindi cha kwanza cha marudio, zoezi la 1: **Hadithi ya Koku na Muta**

Maelekezo ya zana: Bango hili lina kurasa saba zenye picha ya mvulana na msichana wakifanya mazungumzo. Mvulana amebeba ngao na msichana anamuuliza kwanini ameibeba.

Shughuli za kufanya:

- Tumia stendi ya kutundikia kuonyesha bango lenye hadithi ya Koku na Muta mbele ya darasa
- Chagua wanafunzi wawili waje mbele ya darasa, mmoja mvulana na mmoja msichana. Mvulana atasoma sehemu ya Muta na msichana atasoma sehemu ya Koku. Mwambie mvulana anaweza kutumia sauti ya kuchekesha wakati anasoma sehemu ya Muta.

- Katika kila kurasa, jadili na wanafunzi kilichotokea katika ukurasa huo na inamaanisha nini. Mfano, kwenye kurasa ambayo Koku anasema inachukiza Muta hatumii sabuni, weka ***mkazo kwenye kipengele cha kuchukiza*** na ni kwanini ni jambo baya Muta hanawi mikono yake kwa sabuni na maji baada ya kutoka chooni. Sisitiza kuwa Muta atakuwa anakula uchafu au kinyesi iwapo hatanawa mikono kwa maji na sabuni. Waeleze sababu ya Muta kushika ngao ni kuwa anaogopa kuna magonjwa (*"wavamizi"*) yanaweza kuingia kwenye mwili wake.
- Baada ya hadithi kuisha, wale wanafunzi wawili wanaweza kurudi kukaa. Fanya majadiliano na darasa zima ni namna gani Muta amebadilisha tabia na ni kwanini imemsaidia kiafya.

Kipindi cha kwanza cha marudio, zoezi la 2: **Wimbo wa "Paulo una mikono michafu"**

Maelezo ya shughuli hii*:* Utahitaj kuimba wimbo huu pamoja na darasa zima

- Soma na uzungumzie beti za wimbo huu pamoja na watoto ili kuhakikisha kila mmoja anajua maneno ya wimbo kabla ya kuimba

Paulo usije kucheza na sisi una mikono michafu,

Tutachafuka hata kunuka, una mikono michaafu

Paulo usije kucheza na sisi una mikono michaafu

Hutaki kunawa sabuni ndiyo dawa, una mikono michaafu

Paulo usije kula na sisi una mikono michaafu,

Hutaki kunawa sabuni ndiyo dawa, unamikono michaafu

- Baada ya kuimba wimbo, zungumza na wanafunzi kwanini hakuna mtu anayetaka kucheza na Paulo na namana mabadiliko ya tabia yangeweza kumsaidia kupata marafiki

Kipindi cha kwanza cha marudio, zoezi la 3: Mchezo wa kadi kuhusu kunawa mikono

Malekezo ya zana zitakazotumika kwenye mchezo huu: kadi ngumu yenye gurudumu linalozunguka katikati, kitufe cha kuchezea.

Maelekezo ya kufanya:

- Onyesha kadi moja ya mchezo mbele ya darasa na kisha uwaelekeze wanafunzi namna ya kucheza
- Kila mwanafunzi anapata tokeni tano za mikono safi anapoanza kucheza
- Kila mchezo unaweza kuchezwa na watu watano, Kila mtoto ataweka kitufe cha kuchezea kwenye sanduku lililoandikwa *"anzia hapa"*
- Mtoto mmoja anazungusha gurudumu lililopo katikati ya kadi, gurudumu hilo lina mshale ambao utaonyesha idadi ya visanduku ambavyo atatakiwa kusogea mbele kulingana na namba iliyoainishwa na mshale au kuchukua moja ya kadi zilizopangwa.
- Kama mtoto atachukua kadi, atahitajika aisome na afanye kilichoandikwa upande wa chini

- Muda wowote ambapo mtoto amefika kwenye kisanduku fulani ataongeza au apunguze idadi ya tokeni zanye mikono misafi kulingana na maelekezo ya sanduku husika
- Baada ya mtoto mmoja kumaliza kucheza mtoto anayefuata anacheza
- Mchezo utaendelea hadi watoto wote wafikie kisanduku kilichoandikwa *“mwisho”*
- Baada ya kila mmoja kumaliza kucheza, kila mtoto atahesabu amebakiwa na tokeni ngapi. Yule ambaye amebakiwa na tokeni nyingi ndio mshindi.
- Baada ya makundi yote kumaliza kucheza fanya mazungumzo ya darasa zima kuhusu ni kisanduku kipi kilikuwa kizuri na kipi kilikuwa kibaya kufikia na kwanini

Kipindi cha kwanza cha marudio, zoezi la 4: Mchezo wa namna vimelea vya maradhi husambaa

Maelezo ya vifaa vinavyohitajika katika mchezo huu: Mchezo huu unahitaji mpira na vumbi la chaki

Shughuli za kufanya:

- Baada ya kumaliza kuwaelekeza wanafunzi namna ya kunawa mikono bakini nje ya darasa kwa muda mfupi
- Gawanya wanafunzi kwenye makundi kila kundi liwe na wanafunzi kumi
- Wape kila kikundi mpira mmoja na uwaambie waushike
- Mwombe mwalimu mmoja msaada wa kwenda kwenye kila kundi kisha achukue mpira na kugeukia sehemu ambayo wanafunzi hawatamuona kisha apake vumbi la chaki kwenye mpira
- Waambie wanafunzi warushiane mpira, hakikisha kila mwanafunzi kwenye kikundi ameshika mpira
- Baada ya dakika chache, waambie wanafunzi waangalie mikono yao. Wataona kuwa vumbi la chaki limeenea kwenye kundi zima la watoto.
- Baada ya watoto kuona chaki kwenye mikono yao, zungumzia namna vimelea vinaenea kwa kutumia mfano huo wa chaki, na pia ikiwa mwenzao angeenda chooni na akatoka bila ya kunawa mikono kwa sabuni alama hizo za chaki zingekuwa ni kinyesi ambacho kingeenea kwao wote ***(inachukiza).***

Kipindi cha marudio cha 2

Malengo ya kipindi hiki:

- Kuwakumbusha watoto namna sahihi ya kunawa mikono
- Kuwazoeza kwa mara nyingine watoto mambo yaliyomo kwenye zana za kujifunzia

Mambo muhimu ya kuongelea:

- Vichocheo vya kihisia kama hofu, yenye kuchukiza, makuzi, n.k.
- Nyakati muhimu za kunawa mikono, mfano baada ya kutoka chooni na kabla ya kula
- Hatua sahihi za kunawa mikono

Namna ya kuandaa kipindi:

- Andaa ratiba kuonyesha ni madarasa yapi yatafundishwa katika siku gani, pia panga walimu wawili kwa darasa
- Tenga dakika 40 kwa siku kufundisha kipindi hiki cha kwanza kwa kila darasa
- Pitia zana utakazotumia kufundishia kabla ya kipindi ili uweze kuzielewa na kuweza kuwaelezea wanafunzi na kujibu maswali yote yatakayoulizwa

Shughuli zinazohusika kwa kipindi cha marudio:

1. **Hatua za kunawa mikono**
2. **Bango lenye sehemu mbili linaloonyesha namna minyoo huenezwa**
3. **Mchezo wa chakula kichafu**

Kipindi cha pili cha marudio, zoezi la 1: Bango linaloonyesha hatua za kunawa mikono

Maelezo ya zana: Hili ni bango lenye visanduku vitano vinavyoonyesha hatua za kunawa mikono.

Shughuli za kufanya:

- Tundika bango moja linaloonyesha hatua za kunawa mikono mbele ya darasa na mengine mawili, kila moja kwenye katika kuta mbili za pembeni angalau karibu na katikati ya darasa.
- Elezea kila kisanduku cha bango: kwenye kisanduku cha kwanza mtoto anatoka chooni na kwenda sehemu ya kunawia mikono, kwenye kisanduku cha pili, mtoto analowesha mikono kwa maji na kupaka sabuni kwenye mikono yake, kisanduku cha tatu anasugua mikono yake kwa pamoja, kisanduku cha nne anasugua katikati ya vidole na kwenye kisanduku cha mwisho mtoto anasuuza mikono yake kwa maji safi kutoka kwenye tanki la kunawia mikono. Hakikisha unawaambia watoto wakati wanasugua mikono yao kwa pamoja wanatakiwa kuhesabu hadi kumi.
- Baada ya kumaliza kuelezea hatua zote za kunawa mikono, wafundishe watoto wimbo huu na muuimbe darasa zima:

Sasa ni wakati mzuri kwenda kujisaidia

Baada ya kutoka chooni, tunawe mikono yetu

Tunawe mikono kwa sabuni

Tunawe mikono kwa maji

Mikono yetu iwe safi tuepuke magonjwa

Kabla ya kula chakula tunawe mikono yetu

Kabla ya kula matunda tuyaoshe kwa maji

Mtoto mzuri ukumbuka kunawa mikono yake

baada ya kutoka chooni pia kabla ya kula

- Baada ya kuwafundisha watoto wimbo nendeni nje kwenye sehemu za kunawia mikono
- Waonyeshe watoto namna ya kunawa mikono yao, hakikisha unatumia sabuni, sugua mikono kwa pamoja mpaka uone povu kisha hesabu hadi kumi, kisha suuza mikono yako kwa maji safi
- Baada ya kuwaonyesha watoto namna ya kunawa mikono, waite watoto wachache ili wanawe mikono mbele ya wanafunzi wengine. Hakikisha wananawa kwa usahihi na kisha fanya majadiliano kuelezea namna walivyofanya.

Kipindi cha marudio cha 2, Zoezi la 2: Bango lenye sehemu mbili kuhusu namna minyoo inavyoenezwa

Maelezo ya zana: Unapotazama bango upande wa kushoto unaonyesha mvulana anatoka chooni na kula kitafunwa bila kunawa mikono, upande wa kulia unaonyesha msichana anatoka chooni na kunawa mikono yake kwa maji na sabuni kisha anakula kitafunwa

Shughuli za kufanya

- Tundika bango moja mbele ya darasa na mengine mawili, kila moja kwenye kuta za pembeni. Waulize watoto waeleze ni nini wanakiona katika bango hilo na wanahisije kutokanana wanachokiona.
- Waelezee watoto kuwa kutokana na kula kitafunwa bila kunawa mikono huyu mvulana amekula kinyesi au mkojo ambao ulikuwa kwenye mikono yake alipotoka chooni
- Zungumzia upande ambao mvulana hakunawa mikono yake namna ameshambuliwa na minyoo tumboni mwake, na kwa upande wa msichana ambaye alinawa mikono yake hana minyoo tumboni mwake
- Waulize watoto ni kitu gani cha tofauti huyu mvulana angefanya ili asipate minyoo

Kipindi cha marudio cha 2, Zoezi la 3: Mchezo wa uchafuzi wa chakula

Maelezo ya vifaa: Mchezo huu unahitaji uwe na kitafunwa (mfano; andazi, biskuti) fimbo/kijiti na tope

Maelezo/Mambo ya kufanya:

- Chukua kitafunwa (mfano; andazi, biskuti) kutoka kwa timu ya watafiti halafu, wakusanye wanafunzi kwenye kikundi nje ya darasa
- Chagua wanafunzi wawili waje mbele ya kikundi
- Waambie wanafunzi wang'ate sehemu ya kitafunwa
- Baada ya wanafunzi kula sehemu ya kitafunwa, chukua kijiti na kukipangusa kwenye tope
- Paka kiasi kidogo tu cha tope kwenye kitafunwa
- Waulize watoto kama wanaweza kuona tope, wanatakiwa wasiweze kuona tope kwa sababu umepaka kiasi kidogo sana
- Waulize watoto kama wanataka kula tena sehemu ya hicho kitafunwa
- Pindi wanafunzi wasemapo hapana, waulize ni kwanini hawataki tena kula kile kitafunwa
- Baada ya kupata majibu kutoka Kwa wanafunzi, fanya mazungumzo Na darasa kuwa ingawa sio lazima waone tope ulilopaka kwenye kitafunwa, bado tope lipo kwenye hicho kitafunwa hivyo kakifai kuliwa.

- Elezea kuwa wanafunzi wapotoka chooni bila kunawa mikono kwa maji na sabuni, kitu hicho hicho kitatokea kwenye chakula chao. Kinyesi (tope) hubaki kwenye mikono baada ya kutoka chooni, na iwapo mwanafunzi huyo atashika chakula kinyesi kitakwenda kwenye chakula pia ingawa hakionekani.
