# Supplementary material for: Hand hygiene intervention to optimize helminth infection control: Design and baseline results of Mikono Safi–An ongoing school-based cluster-randomised controlled trial in NW Tanzania
Source: PLoS One. 2020 Dec 9;15(12):e0242240. doi: 10.1371/journal.pone.0242240 (PMC7725373; doi:10.1371/journal.pone.0242240)
Supplement: S5 Appendix — (PDF) [file pone.0242240.s005.pdf]

MIKONO SAFI IMPACT EVALUATION

Participant's ID No|___|/|___||___|/|___|___|___|
(District)(School code)(Participant's)

Read: Thank you for participating in our study. Now I will request your participation while I fill out this questionnaire. The questionnaire has 6 sections and it will take us about 30 minutes to complete. All your answers and information will be kept confidential and I will not write your name on it. Please feel free to give me your honest answers as no one will know this information belongs to you. I will read out a sentence and wait for your response, for some of the questions I will read some answers from which you can choose. You are also free to choose not to respond to some of the questions if you don't what to.

SECTION 1: SOCIO-DEMOGRAPHIC INFORMATION

No	Code	Questions and Filters	Coding Categories	Programming notes
101		Stage of research	Baseline survey 1<br>Endline survey 2	
102		Write initials of the three names of the interviewer	|__|__|__|	
103		Write date of interview	|__|__| |__|__|__| |__|__|__|__| Day Month Year	
104		Name of district	Bukoba municipal 1<br>Bukoba rural 2<br>Muleba 3	
105		Name of school	**Bukoba municipality**<br>Ibura 1<br>Nshambya 2<br>Kahororo 3<br>Kiteyagwa 4<br>Bilele 5<br>Lumuli 6<br>Kashenge 7<br>Mgeza mseto 8<br>**Bukoba rural**<br>Iluhya 9<br>Kaishaza 10<br>Kansenene 11<br>Ntoma 12<br>Kanazi 13<br>**Muleba**<br>Rugege 14<br>Omukyaya 15<br>Rwakahoza 16	

No	Code	Questions and Filters	Coding Categories	Programming notes
106		Student's grade	Standard one 1<br>Standard two 2<br>Standard three 3<br>Standard four 4<br>Standard five 5<br>Standard six 6<br>Standard seven 7<br>Others (Specify)_______________ 8	

Read: First, I would like to ask you some general questions about yourself and your family.

No	Code	Questions and Filters	Coding Categories	Programming notes
107		Date of birth	|__|__| |__|__|__| |__|__|__|__| Day Month Year<br>**Write 99 for day or 999 for month or 9999 for year if unknown**	
108		Age	|__|__| Years	Check against the DoB entered above and probe if there is a discrepancy
109		Sex	Male 1<br>Female 2	
110		Who are you currently living with?	Both parents 1<br>Single parent 2<br>Guardian(s) 4<br>Others (Specify) _______________ 3	
111		What does your father or guardian do for a living?	Employed (government/private 1<br>Peasant/farmer/livestock keeper 2<br>Merchant/business/shop/craftsman 3<br>Others (specify) _________________ 4<br>I don't know 5<br>N/A 6<br>**N/A if no living father or male guardian**	
112		What does your mother or guardian do for a living?	Employed (government/private 1<br>Peasant/farmer/livestock keeper 2<br>Merchant/business/shop/craftswoman 3<br>Others (specify) _________________ 4<br>I don't know 5<br>N/A 6<br>**N/A if no living mother or female guardian**	

No	Code	Questions and Filters	Coding Categories	Programming notes
113		How many people slept in your home last night?<br>***(Interviewer: Please explain that this question refers to the household in which the student lives, not the building in which more than one family may be staying. Also in some places, people from the same household sleep in more than one building)***	|__|__| (Number)<br>**Write 99 if the number is not known**	
114		Does your family own any of the following possessions?<br>***(Interviewer: Please read and probe each of the items and circle the appropriate response)***	**Yes** **No** **don't know**	
		Farm/land	1 2 3	
		Cow(s)/pig(s)/goat(s)/sheep	1 2 3	
		Chicken(s)/fowl	1 2 3	
		Television	1 2 3	
		Gas/electric cooker	1 2 3	
		Mobile phone(s)	1 2 3	
		Bicycle	1 2 3	
		Motorcycle	1 2 3	
		Vehicle/tractor/boat	1 2 3	

SECTION 2: DEWORMING HISTORY

Read:Now, I am going to ask you some questions relating to worm treatment which children usually receive in schools somewhere else.

No	Code	Questions and Filters	Coding Categories	Programming notes
201		Were you present at the last deworming campaign conducted in this school?	Yes 1<br>No 2<br>I don't remember 3<br>N/A (if the school was not included in a deworming campaign) 4	If the response is No or N/A skip to question 203
202		Did you receive worm treatment during the last deworming campaign at this school?	Yes 1<br>No 2<br>I don't remember 3<br>N/A (if the school was not included in a deworming campaign) 4	
203		Have you ever received worm treatment somewhere else other than here at school?	Yes 1<br>No 2<br>I don't know 3	If the response is No or 'I don't know' skip to section 3
204		When did you receive that treatment the last time? ***(Interview: Please probe for the time the treatment was given***)	Within the last month 1<br>Between last month and a year ago 2<br>Longer than a year ago 3<br>I don't remember 4	
205		Where did you receive that treatment?	Health facility 1<br>Home 2<br>I don't know 3<br>Others (specify) _______________ 4	

SECTION 3: HAND WASHING BEHAVIOUR

Read: I am now going to ask you some questions about your hand washing practices while at home or here at school.

No	Code	Questions and Filters	Coding Categories	Programming notes
301		This is about what you have done today before this interview. Have you washed your hands at some point today?	Yes 1<br>No 2<br>I don't remember 3	If the responses is No or 'don't remember' skip to question 303
302		How many times did you wash your hands today?	|__|__| **Write 99 if the frequency is not known**	
303		How about yesterday: did you wash your hands yesterday at some time?	Yes 1<br>No 2<br>I don't remember 3	If the response is No or don't remember skip to question 305
304		For the whole of yesterday, how many times did you wash your hands?	|__|__| **Write 99 if the frequency is notknown**	
305		Now I request you to think about the last time you washed your hands.<br><br>When did you wash your hands for the last time?	|__|__| |__|__| |__|__|__|__| Day Month Year<br><br>**Write 99 for day or 999 for month or 9999 for year if unknown**	
306		Now I request you to think about the last time you washed your hands. What were the reasons which made you to wash your hands?<br>***(Interviewer: Please don't prompt, record spontaneous answers)***	Yes No<br>I had just visited the toilet 1 2<br>I washed them before eating 1 2<br>I was told to wash them 1 2<br>My hands were dirty 1 2<br>I don't remember 1 2<br>Others 1 2<br>(specify__________________	

307		The last you washed your hands what did you use to wash them?<br><br>***(Interviewer: Please don't prompt, record spontaneous answer)***	Water only 1<br>Water and soap 2<br>I don't remember 3<br>Other 4<br><br>(specify)_______________	
308		The last you washed your hands where did you wash your hands?<br><br>***(Interviewer: Please don't prompt, record spontaneous answer)***	Home 1<br>School 2<br>I don't remember 3<br>Another location 4<br><br>(specify)_______________	
309		Now I will ask you about your usual practice. When do you usually wash your hands?<br><br>***(Interviewer: Please don't prompt, record all spontaneous response(s)***	Yes No<br>Before eating 1 2<br>After visiting the toilet 1 2<br>I don't know 1 2<br>Others 1 2<br><br>(specify)_______________	
310		In the past few days, were there any occasions when you wanted to wash your hands but you could not?	Yes 1<br>No 2<br>I don't know 3	If the response is No or 'don't know' skip to question 401
311		Think about the last time when you wanted to wash your hands but could not: why did you want to wash hands at the time?	I visited the toilet 1<br>I wanted to eat 2<br>I was told to wash them 3<br>My hands were dirty 4<br>I don't know 5<br>Others 6<br><br>(specify) ___________	
312		What prevented you to wash your hands in that particular instance?	I was in hurry 1<br>I forgot 2<br>There was no water 3<br>There was no soap 4<br>I don't know 5<br>Others 6<br><br>(specify) ___________	

SECTION 4: LATRINE FACILITY AT HOME

Read: Now I am going to ask you some questions about the latrine you use at home. These questions mainly involve what materials your family latrineis made out of and how it works.

No	Code	Questions and Filters	Coding Categories	Programming notes
401		Do you have a latrine at home? (at the house where you live)	Yes 1<br>No 2<br>I don't know 3	If the response is No skip to section 5
402		Can you describe the type of latrine that you use at home?<br>***(Interviewer: Please read each sentence below and circle the responses, prompt for each of them)***<br><br>Does it use flushed water?<br><br>Does it have a hole in the floor?<br><br>Does it have a hole in the floor and a lid to cover the hole?<br><br>Does it have a ventilation pipe?	**Yes No I don't know**<br><br>1 23<br><br>1 2 3<br><br>1 2 3<br><br>1 2 3	
403		What is the wall material of your family latrine?	Bricks 1<br>Metal 2<br>Wood 3<br>Earth/natural materials 4<br>I don't know 5<br>Others 6<br><br>(Specify)_______________	
404		What is the roof material of your family latrine?	Metal 1<br>Grass/natural materials 2<br>No roof 3<br>I don't know 4<br>Others 5<br><br>(Specify)_______________	

No	Code	Questions and Filters	Coding Categories	Programming notes
405		What is the floor material of your family latrine?	Cement 1<br>Wood/natural materials 2<br>Earth/marram/sand 3<br>Others 4<br><br>(Specify) _______________	

SECTION 5: FOOD AND WATER HANDLING AT HOME

Read:I will now ask you some questions about the way your family prepares food and how you use water at home.

No	Code	Question and filters	Coding categories	Programming notes
501		Where do you mainly get the water from that you use at home?<br><br>**(*Interviewer: Don't prompt, circle all spontaneous response(s)* given)**	**Yes** **No**<br>Piped water into house 1 2<br>Tap water in the neighborhood 1 2<br>Well 1 2<br>River/stream 1 2<br>Lake 1 2<br>Water vendor 1 2<br>I don't know 1 2<br>Others 1 2<br><br>(specify: ___________________)	
502		At home what do you do to make the drinking water safe?<br><br>**(*Interviewer: Don't prompt, circle all given response(s)*)**	**Yes** **No**<br>Filtering with cloth1 2<br>Use ceramic filter or1 filter candle 2<br>Boiling 1 2<br>Chemical treatment 1 2<br>We use safe tap water 1 2<br>I don't know 1 2<br>Others 1 2<br>(Specify __________________)	

No	Code	Question and filters	Coding categories	Programming notes
503		At home, how does your mother/guardian keep cooked food?	Left uncovered 1<br>In a covered container 2<br>I don't know 3<br>Others 4<br><br>(Specify ___________________)	
504		At home, how do you mainly prepare the left-over food before eating it?<br><br>***(Interviewer: please prompt and circle all given response(s))***	**Yes** **No**<br>Nothing (just eat) 1 2<br><br>Heating/boiling 1 2<br><br>Warming it up 1 2<br><br>I don't know 1 2<br><br>Others 1 2<br>(Specify _____________ __)	

SECTION 6: PERSONAL RISK FACTORS ASSOCIATED WITH STH INFECTION

Read:Lastly, I will ask a few questions about your personal health.

No	Code	Question and filters	Coding categories	Programming notes
601		Have you ever observed worms while you pass stool?	Yes 1<br>No 2<br>I don't remember 3	If the response is No or 'don't remember' skip to question 603
602		When was the last time you passed worms while defecating?<br><br>***(Interviewer: please probe for time of an incidence and not the exact date)***	Within the last week 1<br>Within the last month 2<br>Several months ago/long time ago 3<br>I don't remember 4	

603		From your understanding, how can you describe diarrhoea?	Passing loose/watery stool 1<br>Others (Specify) 2<br>________________<br>I don't know 3	
604		Over the past seven days have you ever had diarrhoea?<br>With diarrhoea I mean watery or very loose stool for more than 2 times a day.	Yes 1<br>No 2<br>I don't know 3	If No or 'don't know' skip to question 606
605		Over the past seven days, on how many days did you have diarrhea (=very loose stool for more than 2 times a day) ?	|__| **Write 9 if the frequency is unknown**	
606		Have you ever eaten soil?	Yes 1<br>No 2<br>I don't know 3	If No or 'don't know' skip to question 607
607		When was the last time you ate soil?<br><br>**(*Interviewer: Please prompt for the timing of the last incidence and not the exact date*)**	This week 1<br>Within the last week 2<br>Within the last month 3<br>Several months ago/long time ago 4<br>I can't remember 5	
608		What did you use to clean your bottom the last time you defecated?<br><br>***(Interviewer: Please don't prompt, circle all spontaneous response(s)***	**Yes** **No**<br>Nothing 1 2<br>Water 1 2<br>Leaves/plant material 1 2<br>Small stones 1 2<br>Garbage paper 1 2<br>Toilet paper 1 2<br>I don't remember 1 2<br>Other 1 2<br>(specify: ________________?	
Thank the participant for his/her participation				
