# Supplementary material for: Hand hygiene intervention to optimize helminth infection control: Design and baseline results of Mikono Safi–An ongoing school-based cluster-randomised controlled trial in NW Tanzania
Source: PLoS One. 2020 Dec 9;15(12):e0242240. doi: 10.1371/journal.pone.0242240 (PMC7725373; doi:10.1371/journal.pone.0242240)
Supplement: S6 Appendix — (PDF) [file pone.0242240.s006.pdf]

MIKONO SAFI IMPACT EVALUATION **Participant's ID No|___|/|___||___|/|___|___|___|**
(District)(School code)(Participant's)

Soma: Asante sana kwa kushiriki kwenye utafiti huu. Kwa sasa naomba ushirikiano wako wakati najaza hili dodoso. Dodoso hili lina sehemu 6 na itatuchukua kama dakika 30 kumaliza. Majibu yote na taarifa utakazonipatia zitakuwa siri na sitaandika jina lako kwenye dodoso hili. Jisikie huru kunipa majibu ya ukweli kwani hakuna mtu atakayejua taarifa hizi zimetoka kwako. Nitasoma kwa sauti sentensi halafu nitasubiria jibu lako, kwa baadhi ya maswali nitasoma majibu pia ambayo utachagua. Uko huru pia kutojibu baadhi ya maswali ukiamua kufanya hivyo.

SECTION 1: SOCIO-DEMOGRAPHIC INFORMATION

No	Code	Questions and Filters	Coding Categories	Programming notes
101		Hatua ya utafiti	Usaili wa awali (Baseline survey) 1<br>Usaili wa mwisho (Endline survey) 2	
102		Andika vifupisho vya majina matatu ya anayehoji	|__|__|__|	
103		Andika tarehe ya mahojiano	|__|__| |__|__|__| |__|__|__|__| Siku Mwezi Mwaka	
104		Jina la wilaya	Bukoba municipal 1<br>Bukoba rural 2<br>Muleba 3	
105		Jina la shule	**Bukoba municipality**<br>Ibura 1<br>Nshambya 2<br>Kahororo 3<br>Kiteyagwa 4<br>Bilele 5<br>Lumuli 6<br>Kashenge 7<br>Mgeza mseto 8<br><br>**Bukoba rural**<br>Iluhya 9<br>Kaishaza 10<br>Kansenene 11<br>Ntoma 12<br>Kanazi 13<br><br>**Muleba**<br>Rugege 14<br>Omukyaya 15<br>Rwakahoza 16	

No	Code	Questions and Filters	Coding Categories	Programming notes
106		Darasa analosoma mwanafunzi	Darasa la awali 0<br>Darasa la kwanza 1<br>Darasa la pili 2<br>Darasa la tatu 3<br>Darasa la nne 4<br>Darasa la tano 5<br>Darasa la sita 6	

Soma: **Kwa kuanza, ningependa kukuuliza maswali ya ujumla kukuhusu wewe na familia yako.**

No	Code	Questions and Filters	Coding Categories	Programming notes
107		Tarehe ya kuzaliwa	|__|__| |__|__|__| |__|__|__|__| Siku Mwezi Mwaka<br>**Andika 99 kwa siku au 999 kwa mwezi au 9999 kwa mwaka ikiwa haijulikani**	
108		Umri	|__|__| Miaka	Linganisha na tarehe ya kuzaliwa hapo juu na uadisi kama kuna tofauti
109		Jinsia	Kiume 1<br>Kike 2	
110		Kwa sasa unaishi na nani?	Wazazi wote wawili 1<br>Mzazi mmoja 2<br>Mlezi 3<br>Wengine (Taja) ________________ 4	
111		Baba au mlezi wako anafanya kazi gani ya kipato?	Mwajiriwa (serikali/binafsi) 1<br>Mkulima/mfugaji 2<br>Biashara/duka/kazi ya ufundi 3<br>Nyingine (Taja) ________________ 4<br>Sijui 5<br>Hana baba au mlezi wa kiume 6	
112		Mama au mlezi wako anafanya kazi gani ya kipato?	Mwajiriwa (serikali/binafsi) 1<br>Mkulima/mfugaji 2<br>Biashara/duka/kazi ya ufundi 3<br>Nyingine (Taja) ____________ 4<br>Sijui 5<br>Hana mama au mlezi wa kike 6	
113		Ni watu wangapi walilala nyumbani kwenu usiku wa jana? ***(Anayehoji: Tafadhali fafanua - hili swali linahusu idadi ya wanafamilia anaoishi nao, wala sio nyumba ambamo familia zaidi ya moja huenda zinaishi pamoja. Pia wakati mwingine watu wa familia moja wanaweza kulala kwenye nyumba zaidi ya moja)***	|__|__| (namba)<br>**Andika 99 ikiwa idadi haifahamiki**	

No	Code	Questions and Filters	Coding Categories			Programming notes
114		Je, familia yenu inamiliki chochote kati ya vitu hivi vifuatavyo? ***(Anayehoji: Tafadhali soma na dadisi kila moja ya mambo haya na uzungushie jibu linalohusika)***	**Ndiyo**	**Hapana**	**Sijui**	
		Shamba/ardhi	1	2	3	
		Ng'ombe/nguruwe/mbuzi/kondoo	1	2	3	
		Kuku/bata/ndege wengine	1	2	3	
		Televisheni	1	2	3	
		Simu ya mkononi	1	2	3	
		Baiskeli	1	2	3	
		Jiko la umeme/gesi	1	2	3	
		Pikipiki	1	2	3	
		Gari/trekta/boti	1	2	3	

SECTION 2: DEWORMING HISTORY

***Soma**: Sasa, ninakwenda kukuuliza maswali yanayohusu matibabu ya minyoo kwa watoto ambayo mara nyingi hutolewa mashuleni au pia wanaweza kuyapata sehemu nyingine.*

No	Code	Questions and Filters	Coding Categories	Programming notes
201		Je, ulikuwepo hapa shuleni siku ya kampeni ya mwisho ya utowaji wa dawa za minyoo kwa wanafunzi?	Ndiyo 1<br>Hapana 2<br>Sikumbuki 3<br>Shule hii haikushiriki kwenye kampeni 4	Kama jibu ni hapana au shule haikushiriki kampeni ruka hadi swali la 203
202		Je, ulipatiwa dawa za minyoo wakati wa kampeni hiyo ya mwisho hapa shuleni?	Ndiyo 1<br>Hapana 2<br>Sikumbuki 3<br>Shule hii haikushiriki kwenye kampeni 4	
203		Umewahi kutibiwa minyoo sehemu nyingine yeyote tofauti na hapa shuleni?	Ndiyo 1<br>Hapana 2<br>Sijui 3	Kama jibu ni hapana au sijui ruka hadi section 3
204		Ni lini ulipata matibabu hayo mara ya mwisho?<br>***(Anayehoji: Tafadhali dadisi kipindi matibabu yalipotolewa)***	Ndani ya mwezi uliopita 1<br>Kati ya mwezi uliopita na mwaka mmoja 2<br>Zaidi ya mwaka mmoja 3<br>Sikumbuki 4	
205		Ni sehemu gani ulipatiwa matibabu hayo?	Hospitali/Kituo cha afya/zahanati 1<br>Nyumbani 2<br>Sijui 3<br>Nyinginezo (taja) ____________ 4	

SECTION 3: HAND WASHING BEHAVIOUR

Soma: Sasa naomba nikuulize maswali yanayohusu mazoea yako ya kunawa mikono ukiwa nyumbani au hapa shuleni

No	Code	Questions and Filters	Coding Categories	Programming notes
301		Swali hili linahusu mambo uliyofanya leo kabla ya haya mahojiano. Je, umenawa mikono yako wakati fulani leo?	Ndiyo 1<br>Hapana 2<br>Sikumbuki 3	Kama jibu ni hapana au sikumbuki ruka hadi swali la 303
302		Je, ni mara ngapi umenawa mikono yako leo?	|__|__| **Andika 99 ikiwa idadi haifahamiki**	
303		Vipi kuhusu jana. Je, ulinawa mikono yako wakati fulani jana?	Ndiyo 1<br>Hapana 2<br>Sikumbuki 3	Kama jibu ni hapana au sikumbuki ruka hadi swali la 305
304		Kwa siku nzima ya jana, ni mara ngapi ulinawa mikono yako?	|__|__| **Andika 99 ikiwa idadi haifahamiki**	
305		Sasa naomba ufikirie mara ya mwisho uliponawa mikono yako.<br><br>Ni lini ulinawa mikono yako kwa mara ya mwisho?	|__|__| |__|__|__| |__|__|__|__| Siku Mwezi Mwaka<br>**Andika 99 kwa siku au 999 kwa mwezi au 9999 kwa mwaka ikiwa haijulikani**	
306		Wakati huu naomba ufikirie mara ya mwisho uliponawa mikono yako. Ni sababu zipi zilikufanya unawe mikono?<br>***(Anayehoji: Tafadhali usidadisi, rekodi majibu ya moja kwa moja)***	**Ndiyo Hapana**<br>Nilikuwa nimetoka chooni 1 2<br>Nilinawa kabla ya kula 1 2<br>Niliambiwa nioshe mikono 1 2<br>Mikono yangu ilikuwa michafu 1 2<br>Sikumbuki 1 2<br>Nyingine 1 2<br>(Taja________________	

307		Mara ya mwisho uliponawa mikono yako, ulitumia nini kunawa mikono?<br><br>***(Anayehoji:Tafadhali usidadisi, rekodi jibu la moja kwa moja)***	Maji peke yake 1<br>Maji na sabuni 2<br>Sikumbuki 3<br>Nyingine 4<br><br>(Taja)________________	
308		Mara ya mwisho kunawa mikono ulikuwa sehemu gani?<br><br>***(Anayehoji: Tafadhali usidadisi, rekodi jibu la moja kwa moja)***	Nyumbani 1<br>Shuleni 2<br>Sikumbuki 3<br>Sehemu nyingine 4<br><br>(Taja)________________	
309		Sasa, nitakuuliza kuhusu mazoea yako ya kawaida. Ni wakati gani huwa unanawa mikono yako?<br><br>***(Anayehoji: Tafadhali usidadisi, rekodi jibu/majibu yote ya moja kwa moja)***	**Ndiyo** **Hapana**<br>Kabla ya kula 1 2<br>Baada ya kutoka chooni 1 2<br>Sijui 1 2<br>Nyingine 1 2<br><br>(Taja)________________	
310		Kwa siku chache zilizopita, kuna wakati ulitaka kunawa mikono yako lakini haukuweza?	Ndiyo 1<br>Hapana 2<br>Sijui 3	Kama jibu ni hapana au sijui ruka hadi sehemu ya 401
311		Fikiria mara ya mwisho ulipotaka kunawa mikono yako lakini haukuweza: Ni kwa nini ulitaka kunawa mikono wakati huo?	Nilitoka chooni 1<br>Nilitaka kula 2<br>Niliambiwa nioshe mikono 3<br>Mikono yangu ilikuwa michafu 4<br>Sijui 5<br>Nyingine 6<br><br>(Taja) ____________	
312		Ni, nini kilikuzuia kunawa mikono yako wakati huo?	Nilikuwa na haraka 1<br>Nilisahau 2<br>Hakukuwa na maji 3<br>Hakukuwa na sabuni 4<br>Sijui 5<br>Nyingine 6<br>(Taja) ____________	

SECTION 4: LATRINE FACILITY AT HOME

Soma: Wakati huu nitakuuliza maswali yanayohusiana na choo mnachotumia nyumbani. Maswali haya yanahusu hasa vifaa vilivyotumika kutengeneza choo cha familia yenu na jinsi kinavyofanya kazi.

No	Code	Questions and Filters	Coding Categories	Programming notes
401		Je, mna choo nyumbani unapoishi? (kwenye nyumba unayoishi)	Ndiyo 1<br>Hapana 2<br>Sijui 3	Kama jibu ni hapana au sijui ruka hadi section 5
402		Unaweza kunielezea ni aina gani ya choo mnachotumia nyumbani? ***(Anayehoji: Tafadhali soma na dadisi kila moja ya sentensi hizi na uzungushie jibu husika)***	**Ndiyo** **Hapana** **Sijui**	
		Je, kinatumia maji ya kuflashi?	1 2 3	
		Je, kina tundu kwenye sakafu?	1 2 3	
		Je, kina tundu kwenye sakafu na mfuniko wa kufunika tundu?	1 2 3	
		Je kina bomba la kutolea harufu?	1 2 3	
403		Ni vifaa gani vilivyotumika kutengeneza kuta za choo cha nyumbani unapoishi?	Matofali 1<br>Mabati 2<br>Mbao 3<br>Udongo/nyasi/makuti 4<br>Sijui 5<br>Nyingine 6<br><br>(Taja)______________	
404		Ni vifaa gani vilivyotumika kutengeneza paa la choo cha nyumbani unapoishi?	Mabati 1<br>Nyasi/makuti 2<br>Hakuna paa 3<br>Sijui 4<br>Nyingine 5<br><br>(Taja)______________	

No	Code	Questions and Filters	Coding Categories	Programming notes
405		Ni vifaa gani vilivyotumika kutengeneza sakafu ya choo cha nyumbani unapoishi?	Saruji 1<br>Mbao/miti 2<br>Udongo/mchanga/kokoto 3<br>Nyingine 4<br><br>(Taja) _______________	

SECTION 5: FOOD AND WATER HANDLING AT HOME

Soma: Sasa nitakuuliza maswali kadhaa kuhusu namna mnavyoandaa chakula na kutumia maji nyumbani.

No	Code	Question and filters	Coding categories	Programming notes
501		Ni kutoka chanzo gani mnapata maji mnayotumia mara nyingi nyumbani?<br><br>***(Anayehoji: Tafadhali usidadisi, zungushia jibu/majibu yote ya moja kwa moja)***	**Ndiyo Hapana**<br>Mfumo wa maji ya bomba ndani ya nyumba 1 2<br>bomba la maji mtaani 1 2<br>Kisima 1 2<br>Mto/chemchemi 1 2<br>Ziwa 1 2<br>Mfanyabiashara ya maji 1 2<br>Sijui 1 2<br>Chanzo kingine 1 2<br><br>(Taja: ___________________)	
502		Nyumbani, huwa mnafanya nini ili kuyafanya maji ya kunywa yawe salama?<br><br>***(Anayehoji: Tafadhali usidadisi, zungushia jibu/majibu yote yaliyotolewa)***	**Ndiyo Hapana**<br>Kuchuja kwa kitambaa 1 2<br>Kutumia chombo maalum cha kuchuja maji 1 2<br>Kuchemsha 1 2<br>Kuweka dawa 1 2<br>Tunatumia maji salama ya bomba 1 2<br>Sijui 1 2<br>Nyingine 1 2<br><br>(Taja ___________________)	

No	Code	Question and filters	Coding categories	Programming notes
503		Je, hapo nyumbani mama au mlezi wako huwa anahifadhi namna gani chakula akishapika?	kinaachwa wazi 1<br>Chombo chenye mfuniko 2<br>Sijui 3<br>Nyingine 4<br><br>(Taja ___________________)	
504		Je, hapo nyumbani huwa mnaandaa jinsi gani chakula kilicholala kabla ya kula?<br><br>***(Anayehoji: Tafadhali dadisi na uzungushie majibu yote yaliyotolewa)***	**Ndiyo** **Hapana**<br>Hatufanyi kitu (tunakula tu) 1 2<br><br>Kupika/kuchemsha 1 2<br><br>Kupasha joto 1 2<br><br>Sijui 1 2<br><br>Nyingine 1 2<br><br>(Taja______________ __)	

SECTION 6: PERSONAL RISK FACTORS ASSOCIATED WITH STH INFECTION

Soma: Katika sehemu hii ya mwisho, nitakuuliza maswali machache kuhusu afya yako binafsi.

No	Code	Question and filters	Coding categories	Programming notes
601		Je, kuna wakati umewahi kuona umetokwa minyoo wakati unajisaidia haja kubwa?	Ndio 1<br>Hapana 2<br>Sikumbuki 3	Ikiwa jibu ni hapana au sikumbuki ruka hadi swali la 603
602		Ni lini mara ya mwisho uliona umetokwa minyoo wakati ukijisaidia haja kubwa?<br><br>***(Anayehoji: Tafadhali dadisi Kipindi cha tukio na sio tarehe husika)***	Ndani ya wiki moja iliyopita 1<br>Ndani ya mwezi mmoja uliopita 2<br>Miezi mingi iliyopita/muda mrefu uliopita 3<br>Sikumbuki 4	
603		Kwa uelewa wako, unaweza kusema kuharisha maana yake ni nini?	Kupata choo laini sana/cha maji maji 1<br>Nyinginezo (taja) 2<br>________________<br>Sijui 3	
604		Katika kipindi cha siku saba zilizopita, umewahi kuharisha?<br><br>Ninaposema Kuharisha namaanisha kupata choo laini sana au cha maji maji zaidi ya mara 2 kwa siku.	Ndio 1<br>Hapana 2<br>Sijui 3	Kama jibu ni hapana au sijui ruka hadi swali la 606
605		Ndani ya kipindi cha siku saba zilizopita, ni siku ngapi kati ya hizo uliharisha?<br><br>(=kupata choo laini sana au cha maji maji zaidi ya mara 2 kwa siku) ?	|___| **Andika 9 kama idadi haifahamiki**	

No	Code	Question and filters	Coding categories	Programming notes
606		Je, umewahi kula udongo?	Ndio 1<br>Hapana 2<br>Sijui 3	Kama jibu ni hapana au sijui ruka hadi swali la 608
607		Ni lini mara ya mwisho ulikula udongo?<br><br>***(Anayehoji: Tafadhali dadisi kipindi cha tukio la mwisho na sio tarehe husika)***	Wiki hii 1<br>Ndani ya wiki iliyopita 2<br>Ndani ya mwezi uliopita 3<br>Miezi mingi/muda mrefu umepita 4<br>Sikumbuki 5	
608		Ulitumia kitu gani kutawaza mara ya mwisho ulipojisaidia haja kubwa?<br><br>***(Anayehoji: Tafadhali usidadisi, zungushia jibu/majibu yote ya moja kwa moja)***	**Ndiyo** **Hapana**<br>Sikutumia chochote 1 2<br>Maji 1 2<br>Majani/matawi ya miti 1 2<br>Mawe madogo 1 2<br>Karatasi chakavu 1 2<br>Toilet paper 1 2<br>Sikumbuki 1 2<br>Nyingine 1 2<br><br>(specify: ________________?	
Mshukuru mshiriki kwa ushirikiano wake				
