# Supplementary material for: Hand hygiene intervention to optimize helminth infection control: Design and baseline results of Mikono Safi–An ongoing school-based cluster-randomised controlled trial in NW Tanzania
Source: PLoS One. 2020 Dec 9;15(12):e0242240. doi: 10.1371/journal.pone.0242240 (PMC7725373; doi:10.1371/journal.pone.0242240)
Supplement: S7 Appendix — (PDF) [file pone.0242240.s007.pdf]

SCHOOL LEVEL DATA

Read: Thank you for letting us conduct our study in this school. Before talking to students we would like to inquire some general information regarding this school.

SECTION 1: **SCHOOL GENERAL INFROMATION AND DEWORMING HISTORY**

Read: I would like to ask you some few background information of the school including information regarding deworming program done in schools.

No	Code	Questions and Filters	Coding Categories	Programming notes
101		Write initials of the names of the interviewer	|__|__|__|	
102		Write date of interview	|__|__| |__|__|__| |__|__|__|__| Day Month Year	
103		Name of district	Bukoba municipal 1<br>Bukoba rural 2<br>Muleba 3	
104		Name of school	1<br>2<br>3<br>4<br>5<br>6<br>7<br>8<br>9<br>10<br>11<br>12<br>13<br>14	
105		Designation of the respondent	Head teacher 1<br>Assistant head teacher 2<br>Teacher 3<br>Others (Specify) 4<br>______________	

106		Has this school been involved in any deworming campaigns?	Yes 1<br>No 2<br>Not known 3	
107		When was the last time a deworming campaign was done at this school?	|__|__| |__|__|__| |__|__|__|__| Day Month Year<br>**Write 99 for day or 999 for month or 9999 for year if unknown**	
Thank you for your participations				
