# Supplementary material for: Hand hygiene intervention to optimize helminth infection control: Design and baseline results of Mikono Safi–An ongoing school-based cluster-randomised controlled trial in NW Tanzania
Source: PLoS One. 2020 Dec 9;15(12):e0242240. doi: 10.1371/journal.pone.0242240 (PMC7725373; doi:10.1371/journal.pone.0242240)
Supplement: S8 Appendix — (PDF) [file pone.0242240.s008.pdf]

MIKONO SAFI SUB-STUDY ON HAND CONTAMINATION

Participant's ID No|___|/|___||___|/|___|___|___|
(District)(School code)(Participant's)

Read: Thank you agreeing to take part in this sub-study. Now I will request your participation while I fill out this questionnaire. The questionnaire will take us about 10 minutes to complete. All your answers and information will be kept confidential and I will not write your name on it.Please feel free to give me your honest answers as no one will know this information belongs to you. I will read out a sentence and wait for your response, for some of the questions I will read some answers from which you can choose. You are also free to choose not to respond to some of the questions if you don't what to.

SECTION 1: SOCIO-DEMOGRAPHIC INFORMATION

No	Code	Questions and Filters	Coding Categories	Programming notes
101		Write initials of the three names of the interviewer	|__|__|__|	
102		Write date of interview	|__|__| |__|__|__| |__|__|__|__| Day Month Year	
103		Name of district	Bukoba municipal 1<br>Bukoba rural 2<br>Muleba 3	
104		Name of school	Tumaini 1<br>Lumuli 2<br>3<br>4<br>5<br>6<br>7<br>8<br>9<br>10<br>11<br>12<br>13<br>14	
105		Student's grade	Standard one 1<br>Standard two 2<br>Standard three 3<br>Standard four 4<br>Standard five 5<br>Standard six 6<br>Standard seven 7<br>Others (Specify)_______________ 8	

MIKONO SAFI SUB-STUDY ON HAND CONTAMINATION

Participant's ID No|___|/|___||___|/|___|___|___|
(District)(School code)(Participant's)

Read: First, I would like to ask you some general questions about yourself.

No	Code	Questions and Filters	Coding Categories	Programming notes
106		Date of birth	|__|__| |__|__|__| |__|__|__|__| Day Month Year<br>**Write 99 for day or 999 for month or 9999 for year if unknown**	
107		Age	|__|__| Years	Check against the DoB entered above and probe if there is a discrepancy
108		Sex	Male 1<br>Female 2	

SECTION 2: DEWORMING HISTORY

Read:Now, I am going to ask you some questions relating to worm treatment which children usually receive in schools or somewhere else.

No	Code	Questions and Filters	Coding Categories	Programming notes
201		Have you ever received worm treatment here at the school or elsewhere?	Yes 1<br>No 2<br>I don't know 3	If the response is No or 'I don't know' skip to section 3
202		When did you receive that treatment the last time? ***(Interview: Please probe for the time the treatment was given***)	Within the last month 1<br>Between last month and a year ago 2<br>Longer than a year ago 3<br>I don't remember 4<br>5	

MIKONO SAFI SUB-STUDY ON HAND CONTAMINATION

Participant's ID No|___|/|___||___|/|___|___|___|
(District)(School code)(Participant's)

SECTION 3: (i) POSSIBLE SOURCES OF HAND CONTAMINATION.(ii) HANDWASHING BEHAVIOUR TODAY

Read:I am now going to ask you some questions about games you may have played today, about your hand washing practices and when you visited the toilet.

No	Code	Questions and Filters	Coding Categories	Programming notes
301		This is about what you have done today before this interview.<br>Today, did you play any game that involved playing with or touching the soil anywhere in the school premises or elsewhere?	Yes 1<br>No 2<br>I don't remember 3	If the response is No or 'don't remember' skip to question 303
302		After this game did you find time to wash your hands?	Yes 1<br>No 2<br>I don't remember 3	
303		When did you go to the toilet for the last time, to defecate?	Within the last one hour 1<br>Today, but longer ago than 1 hour 2<br>Not yet today 3<br>I do not remember 4	
304		What did you use to clean your bottom the last time you defecated?<br><br>***(Interviewer: Please don't prompt, circle all spontaneous response(s)***	**Yes** **No**<br>Nothing 1 2<br>Water 1 2<br>Leaves/plant material 1 2<br>Small stones 1 2<br>Garbage paper 1 2<br>Toilet paper 1 2<br>I don't remember 1 2<br>Other 1 2<br><br>(specify: ________________?	
305		After going to the toilet to defecate the last time did you find time to wash your hands?	Yes 1<br>No 2<br>I don't remember 3	

MIKONO SAFI <u>SUB-STUDY</u> ON HAND CONTAMINATION

Participant's ID No|___|/|___||___|/|___|___|___|
(District)(School code)(Participant's)

No	Code	Questions and Filters	Coding Categories	Programming notes
306		Have you washed your hands at some point today?	Yes 1<br>No 2<br>I don't remember 3	If the response is No or 'don't remember' skip to question 308
307		How long is this ago?	Within the last one hour 1<br>Between one hour and 3 hours 2<br>Longer ago than 3 hours 3<br>I do not remember 4	
308		The last time you washed your hands what did you use to wash them?<br><br>***(Interviewer: Please don't prompt, record spontaneous answer)***	Water only 1<br>Water and soap 2<br>I don't remember 3<br>Other 4<br><br>(specify)_______________	
309		Have you ever eaten soil?	Yes 1<br>No 2<br>I don't know 3	If No or 'don't know' skip to section 4
310		When was the last time you ate soil?<br><br>**(*Interviewer: Please prompt for the timing of the last incidence and not the exact date*)**	Today 1<br>Sometime during this week 2<br>Longer than a week ago but within 3<br>the last month 4<br>Several months ago/long time ago 5<br>I can't remember 6	

SECTION 4: Collection of hand rinsing specimens

401		***Interviewer: Did the participant rinse his/her hands in the container provided, and did you process it as trained and obtained a water sample for the laboratory?***	Yes 1<br>No 2<br>If No, what was the reason?<br><br>(specify ______________________)	

Interviewer: please thank the student for his/her participation.
