# Supplementary material for: Hand hygiene intervention to optimize helminth infection control: Design and baseline results of Mikono Safi–An ongoing school-based cluster-randomised controlled trial in NW Tanzania
Source: PLoS One. 2020 Dec 9;15(12):e0242240. doi: 10.1371/journal.pone.0242240 (PMC7725373; doi:10.1371/journal.pone.0242240)
Supplement: S9 Appendix — (PDF) [file pone.0242240.s009.pdf]

MIKONO SAFI SUB-STUDY ON HAND CONTAMINATION

Participant's ID No|___|/|___||___|/|___|___|___|
(District)(School code)(Participant's)

Soma: Asante sana kwa kushiriki kwenye utafiti huu mdogo. Kwa sasa naomba ushirikiano wako wakati najaza hili dodoso. Itatuchukua kama dakika 10 kumaliza. Majibu yote na taarifa utakazonipatia zitakuwa siri na sitaandika jina lako kwenye dodoso hili. Tafadhali Jisikie huru kunipa majibu ya ukweli kwani hakuna mtu atakayejua taaarifa hizi zimetoka kwako. Nitasoma kwa sauti sentensi halafu nitasubiria jibu lako, kwa baadhi ya maswali nitasoma majibu pia ambayo utachagua. Uko huru pia kutojibu baadhi ya maswali ukiamua kufanya hivyo.

SECTION 1: SOCIO-DEMOGRAPHIC INFORMATION

No	Code	Questions and Filters	Coding Categories	Programming notes
101		Andika vifupisho vya majina matatu ya anayehoji	|__|__|__|	
102		Andika tarehe ya mahojiano	|__|__| |__|__|__| |__|__|__|__| Siku Mwezi Mwaka	
103		Jina la wilaya	Bukoba municipal 1<br>Bukoba rural 2<br>Muleba 3	
104		Jina la shule	Tumaini 1<br>Lumuli 2<br>3<br>4<br>5<br>6<br>7<br>8<br>9<br>10<br>11<br>12<br>13<br>14	
105		Darasa analosoma mwanafunzi	Darasa la kwanza 1<br>Darasa la pili 2<br>Darasa la tatu 3<br>Darasa la nne 4<br>Darasa la tano 5<br>Darasa la sita 6<br>Darasa la saba 7<br>Nyingine (taja)_______________ 8	

MIKONO SAFI SUB-STUDY ON HAND CONTAMINATION

Participant's ID No|___|/|___||___|/|___|___|___|
(District)(School code)(Participant's)

Soma: Kwa kuanza, ningependa kukuuliza maswali machache ya ujumla kukuhusu wewe

No	Code	Questions and Filters	Coding Categories	Programming notes
106		Tarehe ya kuzaliwa	|__|__| |__|__|__| |__|__|__|__| Siku Mwezi Mwaka<br>**Andika 99 kwa siku au 999 kwa mwezi au 9999 kwa mwaka ikiwa haijulikani**	
107		Umri	|__|__| Miaka	Linganisha na tarehe ya kuzaliwa hapo juu na udadisi kama kuna tofauti
108		Jinsia	Kiume 1<br>Kike 2	

SECTION 2: DEWORMING HISTORY

***Soma**:* Sasa, ninakwenda kukuuliza maswali yanayohusu matibabu ya minyoo kwa watoto ambayo mara nyingi hutolewa mashuleni au pia wanaweza kuyapata sehemu nyingine.

No	Code	Questions and Filters	Coding Categories	Programming notes
201		Je, umewahi kupatiwa matibabu ya minyoo hapa shuleni au sehemu nyingine yeyote?	Ndiyo 1<br>Hapana 2<br>Sijui 3	Kama jibu ni 'hapana' au 'sijui' ruka hadi 'section 3'
202		Ni lini ulipata matibabu hayo mara ya mwisho?<br>***(Anayehoji: Tafadhali dadisi kipindi matibabu yalipotolewa)***	Ndani ya mwezi uliopita 1<br>Kati ya mwezi uliopita na mwaka mmoja 2<br>Zaidi ya mwaka mmoja 3<br>Sikumbuki 4	

SECTION 3: (i) POSSIBLE SOURCES OF HAND CONTAMINATION.(ii) HANDWASHING BEHAVIOUR TODAY

Soma: Sasa nitakuuliza maswali Fulani kuhusu michezo ambayo huenda umecheza leo, pia kuhusu mazoea yako ya kunawa mikono na mara ya mwisho ulipoenda chooni.

No	Code	Questions and Filters	Coding Categories	Programming notes
301		Swali hili linahusu mambo uliyofanya leo kabla ya mahojiano yetu haya. Kwa siku ya leo, umecheza mchezo wowote unaohusisha kushika au kuchezea udongo sehemu yeyote hapa shuleni au kwingine?	Ndiyo 1<br>Hapana 2<br>Sikumbuki 3	Kama jibu ni 'hapana' au 'sikumbuki' ruka hadi swali la 303
302		Baada ya huo mchezo, je uliweza kunawa mikono yako?	Ndiyo 1<br>Hapana 2<br>Sikumbuki 3	
303		Ni lini ulikwenda chooni mara ya mwisho kujisaidia haja kubwa?	Ndani ya saa moja lililopita 1<br>Leo, lakini zaidi ya saa 1 lililopita 2<br>Bado sijakwenda leo 3<br>Sikumbuki 4	
304		Ulitumia kitu gani kutawaza mara ya mwisho ulipojisaidia haja kubwa?<br><br>***(Anayehoji: Tafadhali usidadisi, zungushia jibu/majibu yote ya moja kwa moja)***	**Ndiyo** **Hapana**<br>Sikutumia chochote 1 2<br>Maji 1 2<br>Majani/matawi ya miti 1 2<br>Mawe madogo 1 2<br>Karatasi chakavu 1 2<br>Toilet paper 1 2<br>Sikumbuki 1 2<br>Nyingine 1 2<br>(taja: ________________?	
305		Baada ya kwenda chooni mara ya mwisho kujisaidia haja kubwa, je uliweza kunawa mikono yako?	Ndiyo 1<br>Hapana 2<br>Sikumbuki 3	

MIKONO SAFI SUB-STUDY ON HAND CONTAMINATION

Participant's ID No|___|/|___||___|/|___|___|___|
(District)(School code)(Participant's)

No	Code	Questions and Filters	Coding Categories	Programming notes
306		Je, umenawa mikono yako wakati fulani leo?	Ndiyo 1<br>Hapana 2<br>Sikumbuki 3	Kama jibu ni Hapana au Sikumbuki ruka hadi swali la 308
307		Ni muda gani umepita tangu umenawa mikono?	Ndani ya saa moja 1<br>Kati ya saa moja na masaa matatu 2<br>Zaidi ya masaa matatu 3<br>sikumbuki 4	
308		Mara ya mwisho uliponawa mikono yako, ulitumia nini kunawa mikono?<br><br>***(Anayehoji:Tafadhali usidadisi, rekodi jibu la moja kwa moja)***	Maji peke yake 1<br>Maji na sabuni 2<br>Sikumbuki 3<br>Nyingine 4<br><br>(taja)________________	
309		Je, umewahi kula udongo?	Ndio 1<br>Hapana 2<br>Sijui 3	Kama hapana au sijui ruka hadi section 4
310		Ni lini mara ya mwisho ulikula udongo?<br><br>***(Anayehoji: Tafadhali dadisi kipindi cha tukio la mwisho na sio tarehe husika)***	Leo 1<br>Wakati Fulani ndani ya wiki hii 2<br>Muda mrefu zaidi ya wiki moja lakini ndani ya mwezi uliopita 3<br>Miezi mingi iliyopita/ muda mrefu uliopita 4<br>Sikumbuki 5	

SECTION 4: Collection of hand rinsing specimens

401		***Anayehoji: Je, mshiriki aliosha mikono yake katika chombo chenye maji alichopewa, na je, uliyatayarisha maji hayo kama ulivyoelekezwa na kuchukua sampuli ya kupeleka maabara?***	Ndiyo 1<br>Hapana 2<br>Kama hapana, sababu ni nini?<br><br>(taja _______________________)	

Anayehoji: Tafadhali mshukuru mwanafunzi kwa ushirikiano wake.
